# Polyanionic Cyclodextrin Induced Supramolecular Nanoparticle

**DOI:** 10.1038/s41598-016-0026-z

**Published:** 2016-12-23

**Authors:** He-Lue Sun, Ying-Ming Zhang, Yong Chen, Yu Liu

**Affiliations:** 1 Department of Chemistry, State Key Laboratory of Elemento-Organic Chemistry, Nankai University, Tianjin, 300071 P. R. China; 2 Collaborative Innovation Center of Chemical Science and Engineering (Tianjin), Nankai University, Tianjin, 300071 P. R. China

## Abstract

Ionizable cyclodextrins have attracted increasing attention in host–guest chemistry and pharmaceutical industry, mainly due to the introduction of favorable electrostatic interactions. The ionizable cyclodextrins could not only enhance its own solubility but also induce oppositely charged guests to form more stable complex. However, the aggregation induced by charged cyclodextrins has rarely been reported. In this work, guided by the concept of molecular-induced aggregation, a series of carboxyl modified cyclodextrins were synthesized via “click” and hydrolysis reaction. Then, UV-vis spectrum was used to investigate the aggregating behaviors induced by these cyclodextrins towards the cationic guest molecules. The results showed that only the hepta-carboxyl-β-cyclodextrin could induce the guest molecules to self-assemble into supramolecular spherical nanoparticles. Meanwhile, it could form stable inclusion complex with amantadine, a drug for anti-Parkinson and antiviral. The assembly behaviors were investigated by dynamic light scattering, scanning electron microscope, atomic force microscope, transmission electron microscope and NMR spectroscopy. The supramolecular nanoparticles induced by hepta-carboxyl-β-CD and its inclusion with amantadine could be used to encapsulate the model drug and achieve its controlled releasing behaviors.

## Introduction

**Figure 1:**
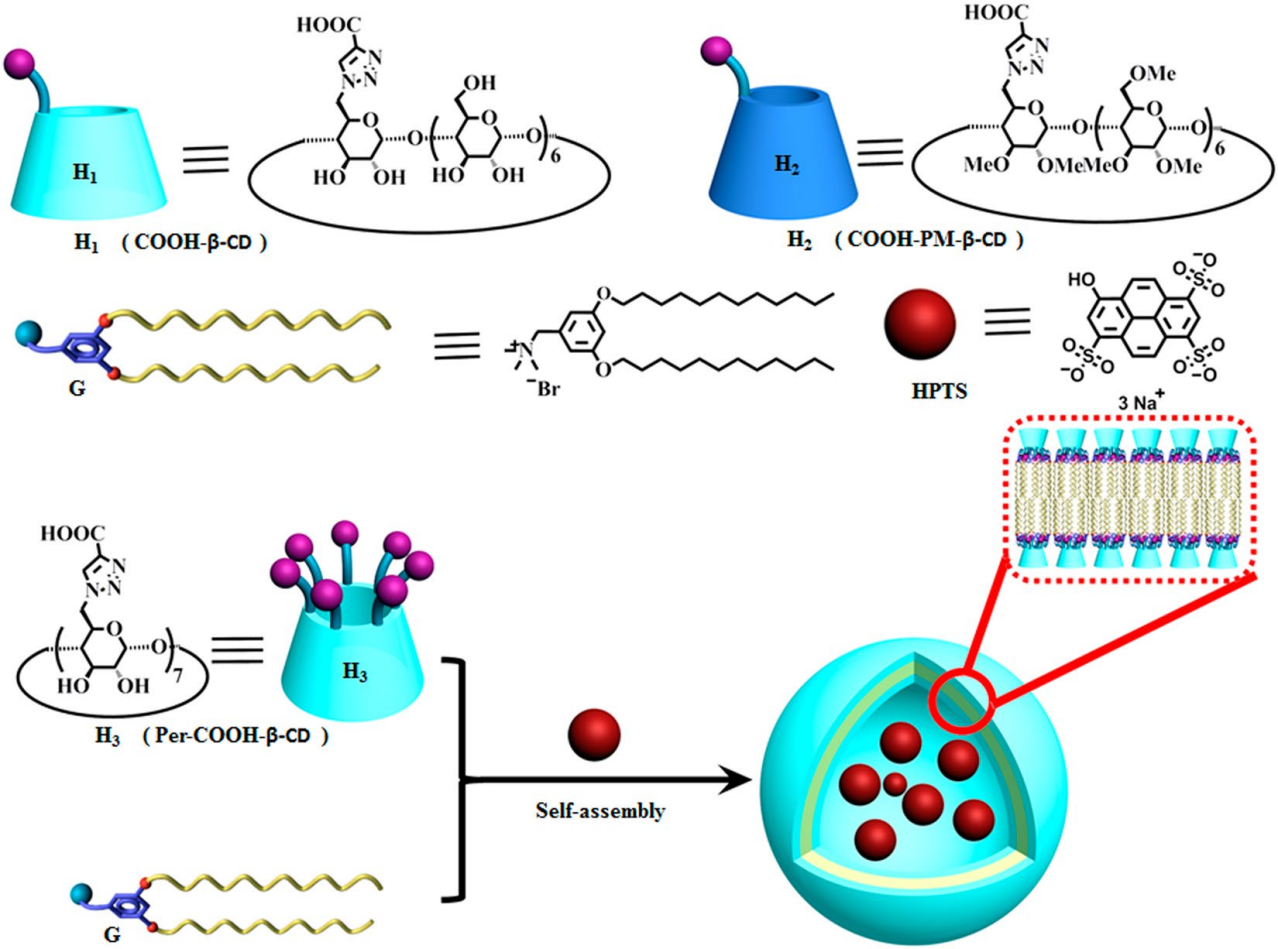
Chemical Structures and Construction of H@G Supramolecular Nanoparticle.

Cyclodextrins (CDs) belong to a class of torus-shaped cyclic oligosaccharides generated from the process of enzymatic degradation, which are nontoxic and commercially available at a relatively low cost. The most investigated CDs are usually with six to eight D-glucose units linked by α-1,4-glucose bonds, referred to as α-, β-, γ-cyclodextrin respectively. Capable of including various shape-compatible organic molecules into their hydrophobic cavities to form inclusion complexes, CDs are widely applied in food, cosmetic, pharmaceutical and diagnostic industries^1–3^. However, the low stability of the inclusion complexes limits the further application of native CDs. Attempting to solve this problem, several strategies have been proposed^4,5^, such as (i) enlargement of the cavity^6^, (ii) hydrogen bonding^7^, (iii) coordination bonding^8^, (iv) addition of electrostatic interaction^9^. Specifically, with the introduction of coulomb interaction, the ionic CDs could not only improve its own solubility but also form highly stable complex with oppositely charged guest molecules^10,11^. This property leads to the wide applications of the ionizable CDs, such as gene^12–14^ and drug delivery^15–17^, separation technology^18,19^, and pharmaceutical^9,20^. Recently, molecular induced aggregation has become an important tool for water soluble macrocycle host molecules, including cucurbiturils^21,22^, sulfonatocalixarenes^23,24^, water-soluble pillararene^25–28^, and cyclodextrins^29–32^, to construct functional supramolecular assemblies. Meanwhile, it’s our special interest to establish a feasible and convenient way to construct the ordered supramolecular nanostructure with various modified cyclodextrins. In the present work, a series of carboxyl modified cyclodextrins (namely **H**_**1–3**_ as shown in Fig. 1 and Fig. S1) were synthesized via “click” and hydrolysis reaction. The induced aggregation behaviors towards the cationic guest molecule **G** were investigated via UV-vis, DLS, SEM, AFM, TEM and NMR spectroscopy. Only the hepta-carboxyl cyclodextrin (**H**_**3**_) can induce the G aggregate into nanoparticle below its critical aggregation concentration (CAC), indicating the multi-charge plays a key role in molecular induced aggregation.

## Results and Discussion

**Figure 2:**
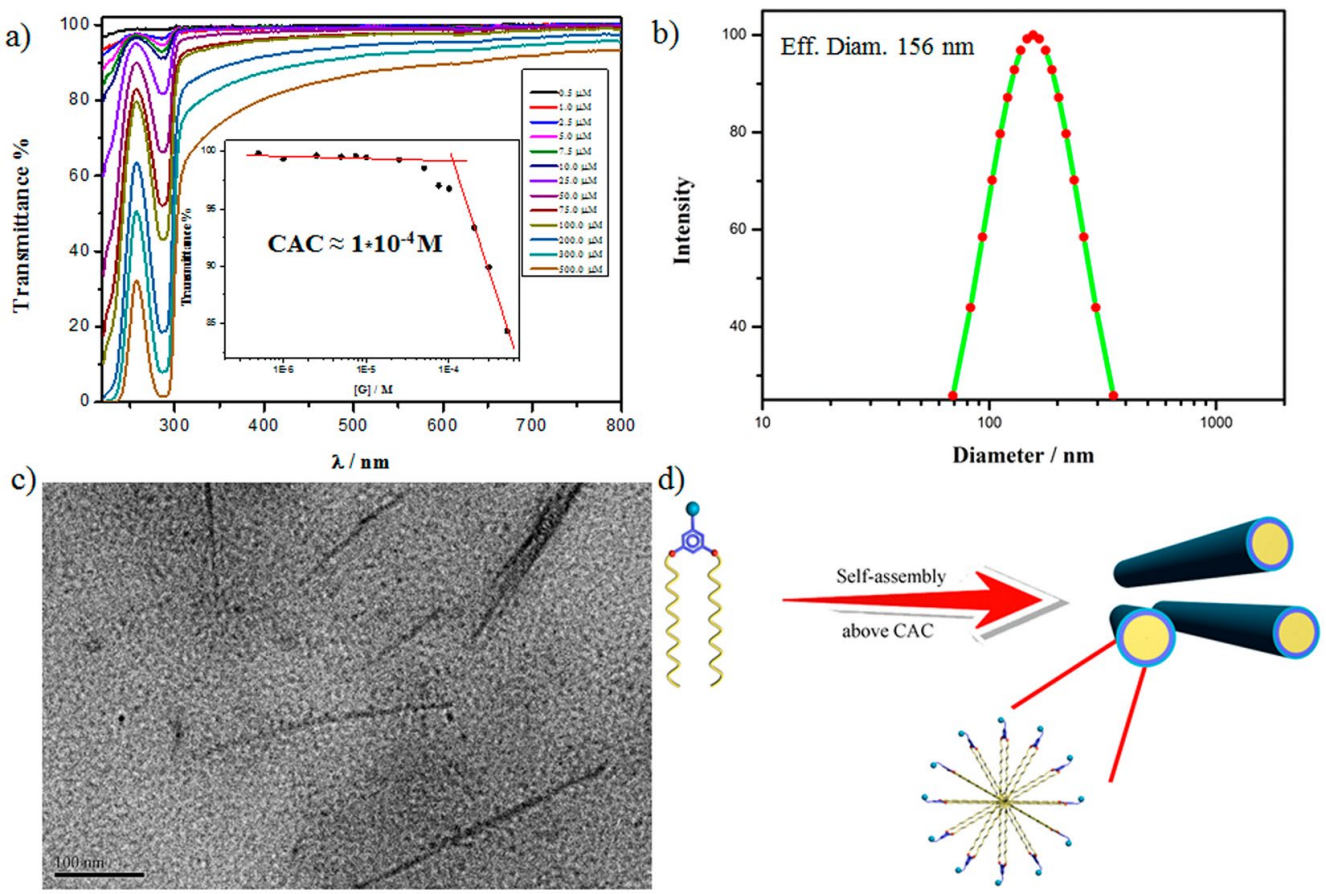
(**a**) Optical transmittance of aqueous solutions of **G**, insert dependence of T_450_% versus [**G**]; (**b**) DLS of **G** above CAC; (**c**) TEM of **G** above CAC; (**d**) Proposed assembly mechanism of **G** above CAC.

The self-assembly behaviors of amphiphilic guest **G**, which is composed of a hydrophilic quaternary ammonium head and two long hydrophobic alkyl chain tails, was investigated using UV-vis, DLS and TEM. The optical transmittance of aqueous solution of G at 450 nm (T_450_%) decreased with the increased concentration, which suggests that **G** could form self-assembly in aqueous solution (as shown in Fig. 2a). The CAC of **G** was measured to be around 0.1 mM. The DLS data showed that the guest **G** formed large aggregates with an average hydrodynamic diameter of *ca.* 156 nm at 0.49 mM above the CAC (as shown in Fig. 2b). In addition, direct morphological information of the G nanostructure is provided by TEM. As shown in Fig. 2c, TEM images of an air-dried solution of **G** exclusively display a number of rod-like micelles with the length of several hundred nanometers. On the basis of the TEM analysis, the average width of the rod-like micelles is *ca*. 9 nm, which fits the twice of length of **G**. Based on these results, the possible assembly mechanism of **G** above the CAC was proposed, as shown in Fig. 2d.

**Figure 3:**
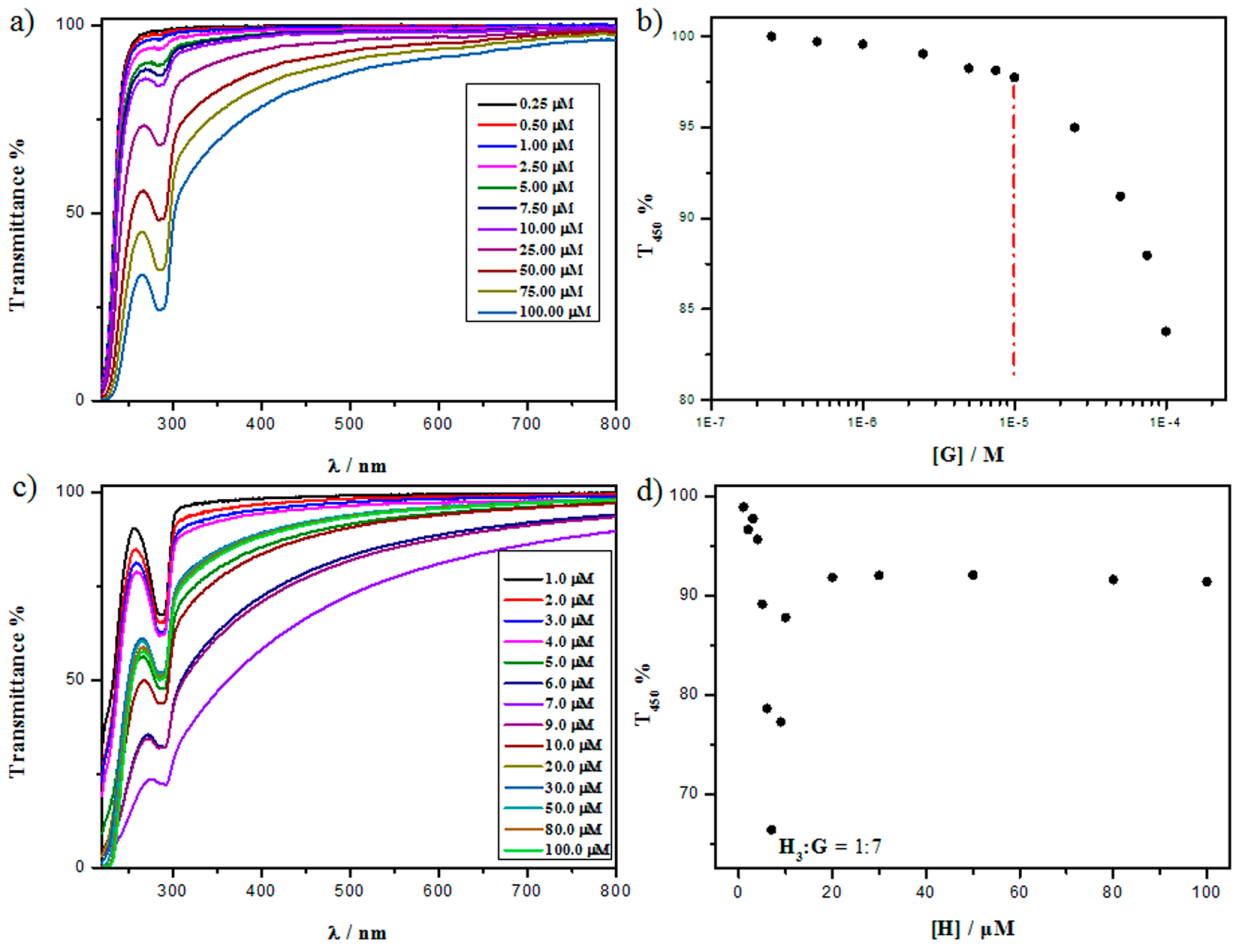
(**a**) Optical transmittance of aqueous solution containing **H**_**3**_ (37.5 μM) and **G** (0.2–100 μM). (**b**) Dependence of T_450%_ versus [**G**]. (**c**) Optical transmittance of aqueous solution containing **G** (49 μM) and **H**_**3**_ (1–100 μM). (**d**) Dependence of T_450_% versus [**H**_**3**_] (pH = 7.0, 25 °C).

The carboxyl modified cyclodextrins (**H**_**1–3**_ in Fig. 1) were obtained via “click” and hydrolysis reaction. To a solution of **G** at 20 μM (which was below the CAC of **G**), an equimolar **H**_**1**_, **H**_**2**_, **H**_**3**_ were added respectively, a significant Tyndall effect was observed only for the complex of **H**_**3**_ and **G**. The corresponding optical transmittance data also confirmed that only the multi-charged cyclodextrin could induce **G** aggregation (Fig. S19). Thereafter, the molecular induced aggregation behavior of **H**_**3**_ was further investigated. As shown in Fig. 3a, with 37.5 μM of **H**_**3**_, the transmittance of complexes was much lower than that of single **G** at the same concentration. According to the plots at T_450_%, the CAC of **G** reduced to *ca.* 10 μM (Fig. 3b). In addition, the preferable mixing ratio between **H**_**3**_ and **G** was determined. The concentration of **G** was fixed at 49 μM, above the CAC of **G** with the presence of **H**_**3**_. The molar ratio of **H**_**3**_ was increased gradually. As shown in Fig. 3c,d, the optical transmittance of the resulting mixture at 450 nm decreased rapidly and then gradually increased and fixed at a stable value, and the minimum was reached at an **H**_**3**_-concentration of 7 μM, referring to **H**_**3**_/**G** ratio of 1:7, *i.e.* ratio of carboxyl anion/quaternary ammonium cation is 1:1. This condition was chosen for the following experiments. The control experiment showed that there was no obvious change for the transmittance of mixture of **G** with **H**_**1**_ and **H**_**2**_, which indicates that at this concentration, neither **H**_**1**_ nor **H**_**2**_ showed molecular induced aggregation behaviors. The results indicate the multi-charge plays an important role in molecular induced aggregation.

**Figure 4:**
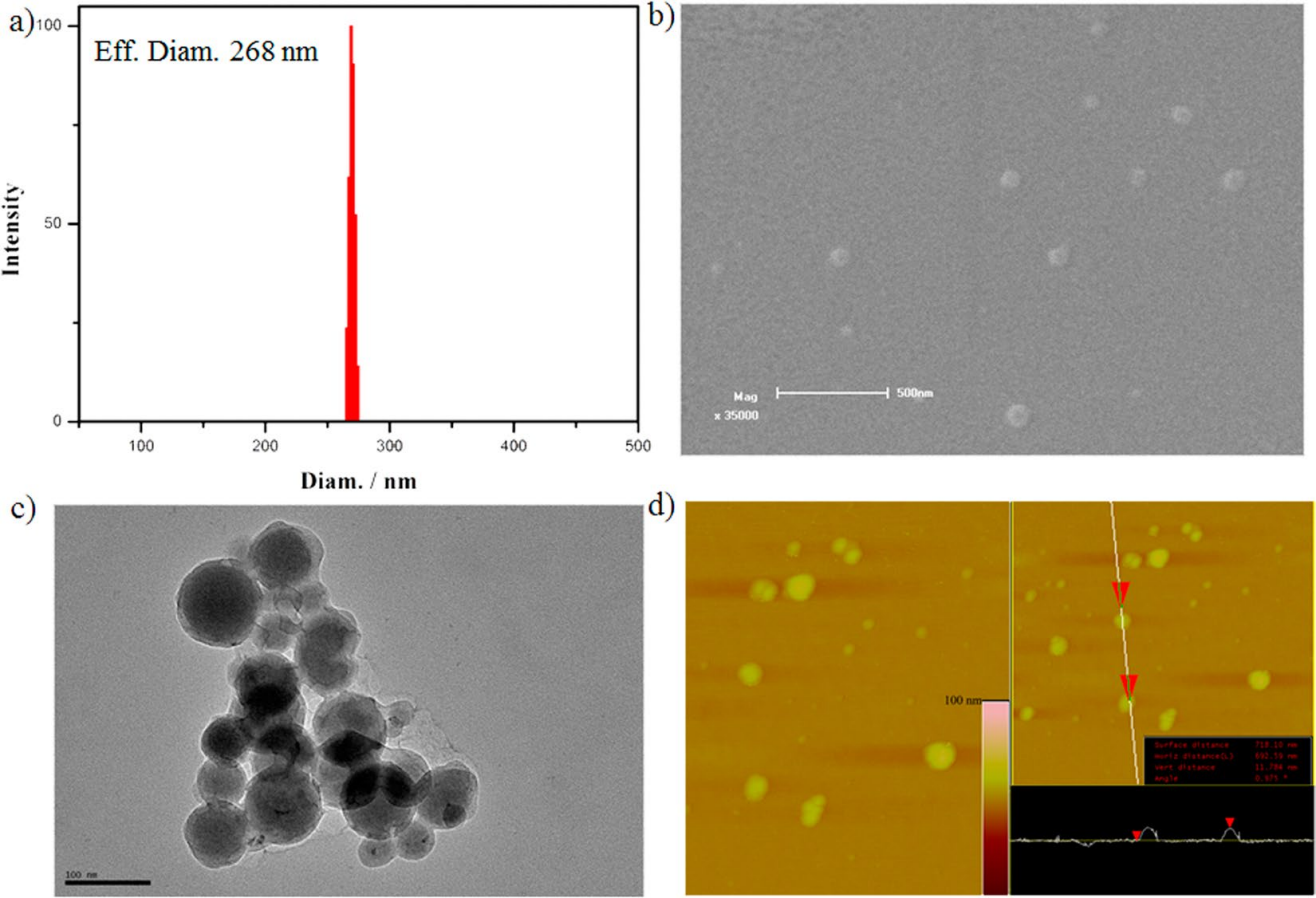
(**a**) DLS (**b**) SEM (**c**) TEM (**d**) AFM of assembly **H**_**3**_@**G**, [**H**_**3**_] = 7 μM, [**G**] = 49 μM, pH = 7.0, 25 °C.

The formation of **H**_**3**_@**G** assembly was further investigated following the determination of the preferable mixing ratio. Since the construction of the assemblies was in an aqueous environment, DLS was employed to investigate the aggregation behaviors in the solution state. As shown in Fig. 4a, the average hydrodynamic diameter was *ca.* 268 nm, which was much larger than that of free G above its CAC, indicating the formation of **H**_**3**_@**G** was diverse to that of free **G**. Furthermore, the morphology of the assembly was investigated by electron microscopies in solid state (Fig. 4b–d). The SEM images showed that it was a spherical nanoparticle with an average diameter of ca. 90 nm, which was smaller than that measured by DLS probably due to the shrinkage of nanoparticles in a drying state during SEM sample preparation. Images produced by TEM and AFM also displayed spherical nanoparticles, and the average diameters of the assemblies fitted well with that measured by SEM. According to the AFM images, the width and height of these **H**_**3**_@**G** assemblies were around 100 and 10 nm, respectively. This observation was probably ascribed to the deformation of these spherical nanoparticles after adsorption onto mica plate, which indicated that the obtained nanoparticles might have a soft core structure^32–34^. However, there was no critical evidence to prove whether such assemblies were vesicles or micelles. Thus, we simply classified them as spherical nanoparticles.

**Figure 5:**
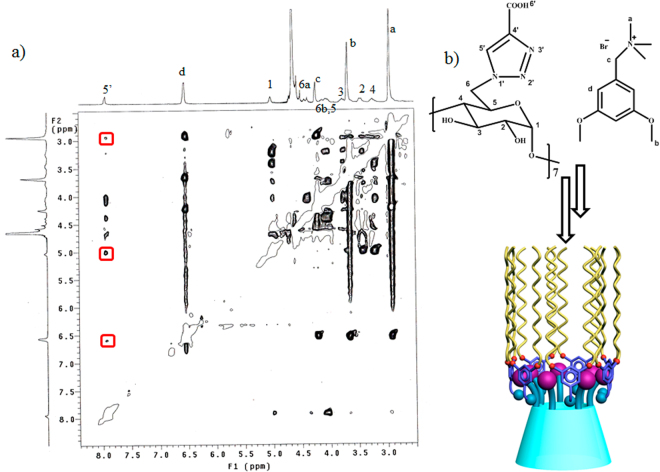
(**a**) 2D ROESY spectrum of **H**_**3**_ **@G**_m_ in D_2_O, 20 °C. (**b**) The proposed aggregation mechanism for **H**_**3**_ **@G**.

After confirming that **H**_**3**_ could induce the **G** aggregation into spherical nanoparticle, the assembly mechanism was investigated. Firstly, NMR spectroscopy was used, as it is a powerful tool to determine molecular structures. According to the 2D ROESY spectrum, there were significant NOE cross peaks for the proton on triazole with the proton 1, 4 and 6a,b on the glucose of CDs, which indicated the carboxyl group cycle around outside of the primary rim of cyclodextrin, as shown in Fig. S11. The circular dichroism experiments were also performed. As shown in Fig. S22, there was a significant negative cotton peak, signing to the absorbance of carboxyl groups, which agreed well with the results from 2D ROESY spectrum. After confirming the configuration of **H**_**3**_, the ^1^H NMR experiment of **H**_**3**_@**G** was performed. Unfortunately, it formed precipitation due to high concentration (Fig. S20). To solve this problem, the reference compound **G**_**m**_ with short chain was synthesized to investigate the assembly behaviors. As shown in Fig. S21, with gradually increased **G**_**m**_, there was a slight shifting to up field for protons on **H**_**3**_. The detail conformation was investigated by 2D ROESY spectrum (Fig. 5a). There were significant NOE cross peaks for proton 5′ with protons a and c on **G**_**m**_. Combining with the preferable mixing ratio for **H**_**3**_ and **G** is 1:7, it indicated that electrostatic interactions played a key role in the molecular induced aggregation. However, there was no cross peak for protons d on **G**_**m**_ with protons on glucose of **H**_**3**_. The results jointly suggested that the **G**_**m**_ was gathered around the **H**_**3**_ not bind into the cavity of **H**_**3**_. Meanwhile, taking the hydrophilicity and hydrophobicity interactions into consideration, the aggregation mechanism would be that the **H**_**3**_, with multi charges, gather the oppositely charged **G** around to form the supramolecular amphiphilic system, such as in Fig. 5b. A further aggregation may take place to form the spherical nanoparticles.

Investigations about the stability of **H**_**3**_@**G** were focused on their tolerance to temperature, time, and pH value via optical transmittance and DLS experiments (Fig. S23). The experiments of tolerance to temperature were performed, during the assembly solution in a glass cell heated from 25 to 70 °C. Surprisingly, there was no appreciable change founded in the transmittance at 450 nm and average efficient diameter. The tolerance to time was investigated at physiological temperature (37 °C), and the transmittance spectra and DLS of **H**_**3**_@**G** were recorded every 30 minutes. As shown in Fig. S23b,e, no obvious change was found in transmittance and average diameter. And also, the same result was found for the tolerance to pH value. According to these results, the **H**_**3**_@**G** nanoparticles showed sufficient stability for further investigation.

**Figure 6:**
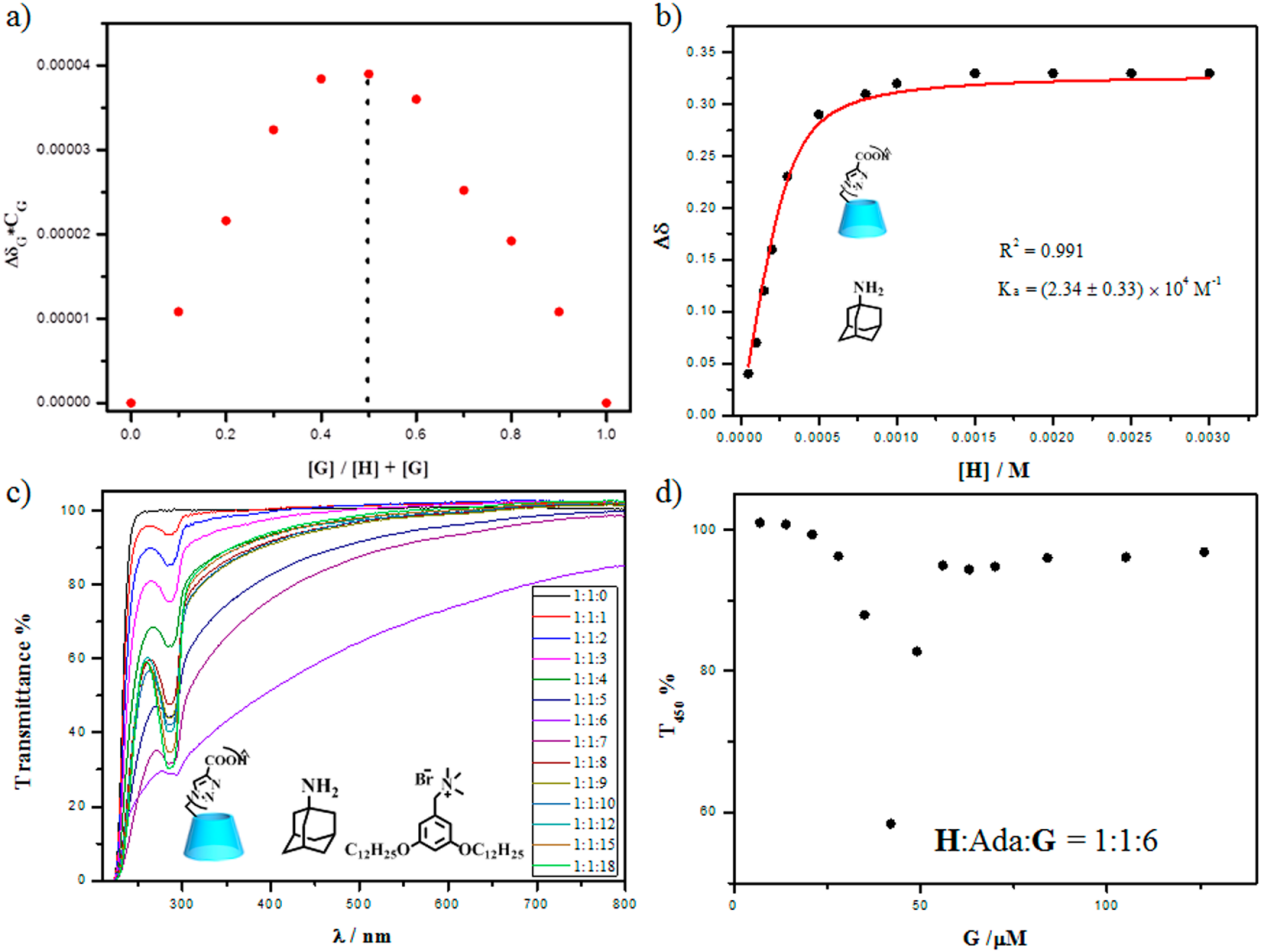
(**a**) Job’s experiment, (**b**) Binding constant of **H**_**3**_ with amantadine measured by NMR spectra in D_2_O, 20 °C. (**c**) Optical transmittance of aqueous solution containing **H**_**3**_ and amantadine ([**H**_**3**_] = [Ama] = 7 μM) and G (0–126 μM). (**d**) Dependence of T_450_% versus [**G**]. pH = 7.0.

Adamantane derivatives are well known as guest molecules for construction supramolecular assemblies due to the ability of forming stable inclusion complex with β-CD^35–38^. Meanwhile, amantadine (Ama), one of the derivatives of adamantane, has been used as pharmacological drugs for anti-Parkinson and antiviral^39^. As mentioned above, **G** was gathered around the **H**_**3**_ not included inside its cavity. Thus, amantadine was chosen as a model drug. Firstly, the binding behaviours toward amantadine to **H**_**3**_ were investigated. The Job analysis of the NMR spectral data gave the complexation stoichiometry binding radio of 1:1 between **H**_**3**_ and Ama, and the apparent binding constant was calculated as 2.34 × 10^4 ^M^−1^ (Fig. 6a,b). The conformation of **H**_**3**_ with amantadine was also investigated by ^1^H NMR and 2D ROESY spectrum. As shown in Fig. S24, with the addition of **H**_**3**_, there was down field shift with the protons on amantadine in different extent, which may be caused by binding into the cavity of **H**_**3**_ to form inclusion complex. Meanwhile, according to the 2D ROESY spectrum, there were significant NOE cross peaks for the protons of amantadine with 3,5-protons on **H**_**3**_ (Fig. S25). The results jointly indicated that the amantadine was bound into the cavity of **H**_**3**_.

Moreover, the preferable mixture ratio of the ternary complex was determined. Taking the previous results into consideration, the concentrations of **H**_**3**_ and amantadine were fixed at 7 μM, and then increased the concentration of **G**. The value of T_450%_ decreased sharply and then increased and fixed at a stable value. The minimum was reached at 42 μM (Fig. 6c,d), referring to ternary complex ratio of 1:1:6, *i.e.* ratio of anion/cation ratio is 1:1. Then the self-assembly behaviors of the ternary complexion at the same concentration was investigated by DLS and TEM. The DLS data showed that the assembly was formed with an average hydrodynamic diameter of ca. 300 nm. And also, the TEM images displayed spherical nanoparticle (Fig. S26).

**Figure 7:**
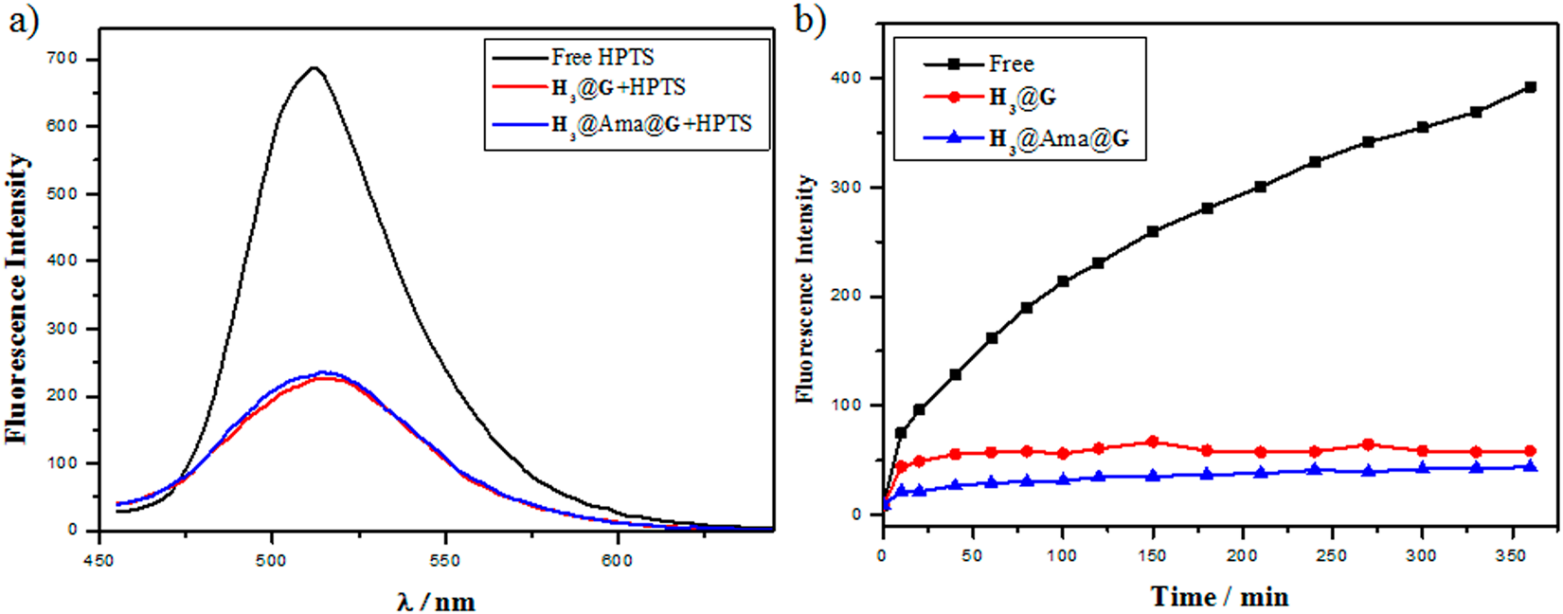
(**a**) Fluorescence emission spectra of free HPTS and HPTS-loaded assembly. (**b**) *In vitro* release profiles of HPTS from free HPTS and HPTS-loaded assembly pH = 7.0, 37 °C.

During the last years, drug delivery and controlled release systems have attracted more and more attention due to the benefits such as therapeutic effects, reduced toxicity, decreased medication time and so on^40–43^. The highly stable nanoparticle that is constructed via molecular induced aggregation may be applied in controlled release. The two different nanoparticles, **H**_**3**_@**G** and **H**_**3**_@Ama@**G**, obtianed in the above experiments might be used in drug delivery and controlled release. To prove this, the drug loading experiments were tested. In the substrate loading experiments, the trisodium salt of 8-hydroxypyrene-1,3,6-trisulfonic acid (HPTS) was selected as a model molecule. The capability of encapsulation and release of the supramolecular nanoparticle were examined by fluorescence spectra, as shown in Fig. 7a. After loading the free HPTS to the nanoparticles, the florescence intensity of HPTS quenched by 67% and 66% for **H**_**3**_@**G** and **H**_**3**_@Ama@**G** respectively, indicating that the model drug HPTS was loaded into the nanoparticles. Further, the release behaviours of the assemblies were investigated. The release profiles of the supramolecular assemblies loaded HPTS and free HPTS in ultrapure water were shown in Fig. 7b. It can be seen that the release rate of free HPTS solution was much significantly faster than that of HPTS-loaded assemblies. More than 80% free HTPS was released in 360 min, but only 10% HTPS was released when protected by supramolecular nanoparticle. These results jointly implied that the drug loading and controlled release could be obtained for the **H**_**3**_@**G** and **H**_**3**_@Ama@**G** assemblies. Therefore, the supramolecular nanoparticles could serve as new nanocapsules to load and release drug agents.

## Conclusions

In conclusion, a series of carboxyl modified cyclodextrins (**H**_**1–3**_) were synthesized via “click” and hydrolysis reaction in order to study their induced aggregation behaviors. Unexpectedly, only the hepta-carboxyl-β-cyclodextrin (**H**_**3**_) can induce the cationic guest molecule **G** aggregate into nanoparticle below its CAC, indicating the multi charged play key roles in molecular induced aggregation. The preferable ratios for mixtures of **H**_**3**_@**G** and **H**_**3**_@Ama@**G** were 1:7 and 1:1:6 respectively, i.e., anion to cation is 1:1. In addition, **H**_**3**_ could include the amantadine, from which the produced nanoparticle could load model drug HPTS and achieve its controlled-release. That is, with the potential capability of combining two different drugs into a supramolecular nanoparticle, it may be used as nanocapsules for clinical application.

## Methods

### Materials

All the reagents and solvents were commercially available and used as received unless otherwise specified purification. Anhydrous *N*,*N*-dimethylformamide (DMF) was dried and distilled over CaH_2_ under reduced pressure. All aqueous solutions were prepared with distilled water, pH = 7.0. β-CD of reagent grade was recrystallized twice from water and dried under vacuum at 95 °C for 24 h prior to use. The 6-deoxy-6-azido-β-CD (**A**_**1**_)^44,45^, 6-deoxy-6-azido-permethyl-β-CD (**A**_**2**_)^45^, per-6-azido-β-CD (**A**_**3**_)^46^, 3,5-bis(dodecyloxy)benzyl bromide (**1**)^47^ and 3,5-dimethoxybenzyl bromide (**2**)^47^ were prepared according to the literature procedure. Column chromatography was performed on silica gel (200–300 mesh).

### Measurements

#### UV/Vis absorption and fluorescence emission spectroscopy

UV/Vis spectra were recorded in a quartz cell (light path 10 mm) equipped with a temperature controller. Steady-state fluorescence spectra were recorded in a conventional quartz cell (light path 10 mm).

#### DLS measurements

The solvent was filtered through a 0.45 mm Millipore filter. The samples were dissolved in the filtered solvent and used without further filtering. A sample solution (2 mL) was poured into a clean scintillation vial. The samples were examined using a laser-light scattering spectrometer equipped with a digital correlator at 636 nm at a scattering angle of θ = 90°.

#### TEM measurements

A 5 μL portion of the dilute aqueous solution was dropped onto a copper grid. Then the grid was air-dried. The samples were examined at an accelerating voltage of 200 keV.

#### AFM measurements

A 25 μL portion of the dilute sample solution was dropped onto a new mica surface. Two minutes later, the excess amount of aqueous solution was blotted away with a piece of filter paper. The mica was washed with distilled water (1 mL) and then air-dried. The samples were examined in tapping mode in the air at room temperature.

#### SEM measurements

A 50 μL portion of the sample solution was dropped onto a coverslip followed by evaporating the liquid in Shimadzu SS-550 SEM operating at an accelerating voltage of 30 keV.

#### Preparation of H_1_, H_2_, H_3_, G and G_m_

Preparation of **H**_**1**_ (COOH-β-CD). To a solution of Methyl propiolate (130 mg, 1.5 mM) in THF (20 ml), 6-deoxyl-6-azido-β-CD (1.16 g, 1 mM) in water (20 mL) was added with stirring. To the resulting solution, the solution of CuSO_4_·5H_2_O (750 mg, 3 mM) and sodium ascorbate (1 g, 5 mM) in water 10 mL was added. Then the mixture solution was heated at about 50 °C for 48 h. The insoluble precipitates were removed by filtration, and the filtrates were dried under reduced pressure. The product was used directly without further purification. The product was added to the solution of KOH (2.2 g, 40 mM) in H_2_O:methanol 1:1 (40 ml), and heated at reflux for 3 h. The reaction mixture was acidified with 1 M HCl to pH 3~4, then dried under reduced pressure. The crude product was further purified by HPLC (reversed phase) with water-acetonitrile (v/v = 80:20) eluent, the collected fraction was freeze-dried to obtain violet powder in 35% yield. ^1^H NMR (400 MHz, D_2_O, ppm): δ = 8.29 (s, 1H), 5.16 (s, 1H), 5.00 (d, J = 29.2 Hz, 7H), 4.68–4.56 (m, 1H), 4.17 (d, J = 8.9 Hz, 1H), 4.03–3.45 (m, 37H), 3.15 (d, J = 12.0 Hz, 1H), 2.78 (d, J = 11.8 Hz, 1H). ^13^C NMR (101 MHz, D_2_O) δ 166.5, 143.5, 129.0, 102.0, 102.0, 101.9, 101.3, 83.0, 81.3, 81.1, 81.1, 80.5, 73.0, 72.7, 72.0, 71.8, 71.7, 71.6, 71.4, 70.5, 60.3, 60.1, 58.9, 51.3. MODI-TOF-MS: *m*/*z*: 1228.31, ([M–H]^+^ Calcd for C_45_H_70_N_3_O_36_^+^, 1228.37).

Preparation of **H**_**2**_ (COOH-PM-β-CD). Almost the same procedures described above were employed. The crude product obtained was further purified by flash column chromatography using a chloroform-methanol (v/v = 35:1) eluent to give the product as a foam powder in 50% yield. ^1^H NMR (400 MHz, D_2_O, ppm): δ = 8.22 (s, 1H), 5.43 (s, 1H), 5.36 (d, J = 3.5 Hz, 1H), 5.29 (d, J = 9.4 Hz, 4H), 5.18 (dd, J = 8.1, 3.1 Hz, 2H), 5.09 (d, J = 12.6 Hz, 1H), 4.60 (dd, J = 14.3, 9.5 Hz, 1H), 4.17 (t, J = 8.9 Hz, 1H), 4.02 (s, 1H), 3.93–3.14 (m, 95H), 3.05 (d, J = 10.8 Hz, 1H), 2.93 (d, J = 8.7 Hz, 1H). ^13^C NMR (101 MHz, D_2_O) δ 166.4, 144.0, 129.3, 97.6, 81.1, 80.9, 80.7, 80.0, 76.3, 70.6, 70.4, 70.1, 69.6, 60.4, 59.9, 59.7, 59.6, 59.3, 58.5, 58.4, 58.4, 58.3, 58.1, 58.1, 58.0, 57.9, 57.8, 51.5. ESI-MS: *m*/*z*: 1532.5, ([M + Na]+ Calcd for C_65_H_111_N_3_O_36_Na+, 1532.68).

Preparation of **H**_**3**_ (Per-COOH-β-CD). Methyl propiolate (1.68 g, 20 mM) was added to a stirred solution of per-6-azide-permethyl-β-CD (1.31 g, 1 mM) in DMF (50 mL), to which CuI (1.9 g, 10 mM,) was added under argon at room temperature. The mixture was stirred at about 60 °C for 48 h. After cooling to room temperature, the mixture was filtered to remove any insoluble copper salt, and 200 ml water was added into the filtrate. The precipitation was filtered and washed with acetone. The crude product was added to the solution of KOH (4.4 g, 80 mM) in H_2_O:methanol 1:1 (80 ml) without further purification, and heated at reflux for 3 h. The reaction mixture was acidified with 1 M HCl to pH 3~4, then dried under reduced pressure. The crude product was further purified by HPLC (reversed phase) with water-acetonitrile (v/v = 85:15) eluent, the collected fraction was freeze-dried to obtain violet powder in 19 % yield. ^1^H NMR (400 MHz, D_2_O, ppm): δ = 8.11 (s, 7H), 5.10 (d, J = 3.5 Hz, 7H), 4.45 (d, J = 12.2 Hz, 7H), 4.26–4.10 (m, 14H), 3.93 (t, J = 9.4 Hz, 7H), 3.53 (dd, J = 10.0, 3.4 Hz, 7H), 3.34 (t, J = 9.1 Hz, 7H). ^13^C NMR (101 MHz, D_2_O) *δ* 166.0, 143.4, 129.3, 101.7, 82.45, 72.3, 71.6, 70.0, 50.3. MODI-TOF-MS: *m*/*z*: 1798.54, ([M–H]^+^ Calcd for C_63_H_76_N_21_O_42_^+^, 1798.45).

Preparation of **G**. To a solution of 3,5-Bis(dodecyloxy)benzyl bromide (538 mg, 1 mM) in toluene (15 ml), excess Trimethylamine alcoholic solution(2.5 ml) was added. Then the mixture solution was heated at reflux overnight. After cooling to room temperature, the mixture was dried under reduce pressure. The crude product was further purified by flash column chromatography using a chloroform-methanol (v/v = 25:1) eluent to give the product as a white viscous solid in 70% yield. ^1^H NMR (400 MHz, CDCl_3_, ppm): δ = 6.71 (s, 2H), 6.54 (s, 1H), 4.86 (s, 2H), 3.94 (s, 4H), 3.42 (s, 9H), 1.75 (s, 4H), 1.62 (s, 4H), 1.43 (s, 4H), 1.26 (s, 28H), 0.88 (s, 6H).^13^C NMR (101 MHz, CDCl_3_) δ 160.8, 128.9, 111.3, 103.2, 69.5, 68.5, 53.1, 31.9, 29.7, 29.6, 29.6, 29.6, 29.4, 29.3, 29.2, 26.1, 22.7, 14.1. ESI-MS: *m*/*z* 518.5, ([G–Br]^+^ Calcd for C_34_H_64_NO_2_^+^, 518.5).

Preparation of **G**_**m**_. Almost the same procedures described above were employed. The crude product obtained was further purified by flash column chromatography using a chloroform-methanol (v/v = 20:1) eluent to give the product as a foam powder in 75% yield. ^1^H NMR (400 MHz, D_2_O, ppm): δ = 6.67 (d, J = 9.8 Hz, 3H), 4.33 (s, 2H), 3.77 (s, 6H), 3.02 (s, 9H). ^13^C NMR (101 MHz, D_2_O) δ 160.5, 129.5, 111.2, 102.3, 69.4, 55.7, 52.6. ESI-MS: *m*/*z*: 210.1, ([G_m_–Br]^+^ Calcd for C_12_H_20_NO_2_^+^, 210.1).

## Electronic supplementary material

Supplementary Information
