# Supplementary material for: Polyanionic Cyclodextrin Induced Supramolecular Nanoparticle
Source: Sci Rep. 2016 Dec 23;6:27. doi: 10.1038/s41598-016-0026-z (PMC5431346; doi:10.1038/s41598-016-0026-z)
Supplement: Supplementary file 1 — Supplementary Information [file 41598_2016_26_MOESM1_ESM.doc]

**Supporting Information for**

**Polyanionic Cyclodextrin Induced Supramolecular Nanoparticle**

He-Lue Sun,^1^ Ying-Ming Zhang,^1^ Yong Chen,^1,2^ and Yu Liu*^,1,2^

^1^Department of Chemistry, State Key Laboratory of Elemento-Organic Chemistry, Nankai University, Tianjin 300071 (P. R. China).

^2^Collaborative Innovation Center of Chemical Science and Engineering (Tianjin), Nankai University, Tianjin 300071 (P. R. China).

^*^Address correspondence to yuliu@nankai.edu.cn

**Table of Contents**

[Figure S1 The synthesis routes of molecule **H_1_, H_2_ H_3_, G,** and **G_m_**. S3](#__RefHeading___Toc458610004)

[Figure S2. ^1^H NMR spectrum of **H_1_** S3](#__RefHeading___Toc458610005)

[Figure S3. ^13^C NMR spectrum of **H_1_** S4](#__RefHeading___Toc458610006)

[Figure S4. MALDI-TOF-MS spectrum of **H_1_** S4](#__RefHeading___Toc458610007)

[Figure S5. ^1^H NMR spectrum of **H_2_** S5](#__RefHeading___Toc458610008)

[Figure S6. ^13^C NMR spectrum of **H_2_** S5](#__RefHeading___Toc458610009)

[Figure S7. ESI-MS spectrum of **H_2_** S6](#__RefHeading___Toc458610010)

[Figure S8. ^1^H NMR spectrum of **H_3_** S6](#__RefHeading___Toc458610011)

[Figure S9. ^13^C NMR spectrum of **H_3_** S7](#__RefHeading___Toc458610012)

[Figure S10. HSQC spectrum of **H_3_** S7](#__RefHeading___Toc458610013)

[Figure S11. 2D ROESY spectrum of **H_3_** S8](#__RefHeading___Toc458610014)

[Figure S12. MALDI-TOF-MS spectrum of **H3** S8](#__RefHeading___Toc458610015)

[Figure S13. ^1^H NMR spectrum of **G** S9](#__RefHeading___Toc458610016)

[Figure S14. ^13^C NMR spectrum of **G** S9](#__RefHeading___Toc458610017)

[Figure S15. ESI-MS spectrum of **G** S10](#__RefHeading___Toc458610018)

[Figure S16. ^1^H NMR spectrum of **G_m_** S10](#__RefHeading___Toc458610019)

[Figure S17. ^13^C NMR spectrum of **G_m_** S11](#__RefHeading___Toc458610020)

[Figure S18. ESI-MS spectrum of **G_m_** S11](#__RefHeading___Toc458610021)

[Figure S19. Tyndall effect and transmittance spectra S12](#__RefHeading___Toc458610022)

[Figure S20. ^1^H NMR of **H_3_**@**G** S12](#__RefHeading___Toc458610023)

[Figure S21. ^1^H NMR of **H_3_** with various ratio of **G_m_** S13](#__RefHeading___Toc458610024)

[Figure S22. UV-Vis and CD spectra S13](#__RefHeading___Toc458610025)

[Figure S23. The stability of the assembly **H_3_**@**G** S14](#__RefHeading___Toc458610026)

[Figure S24. ^1^H NMR spectra of **H_3_, Ama and H_3_@**Ama S14](#__RefHeading___Toc458610027)

[Figure S25. 2D ROESY spectrum of **H_3_**@Ama S15](#__RefHeading___Toc458610028)

[Figure S26. DLS and TEM image of **H_3_**@Ama@**G** S15](#__RefHeading___Toc458610029)

Figure S1. The synthesis routes of molecule **H_1_, H_2_ H_3_, G,** and **G_m_.**


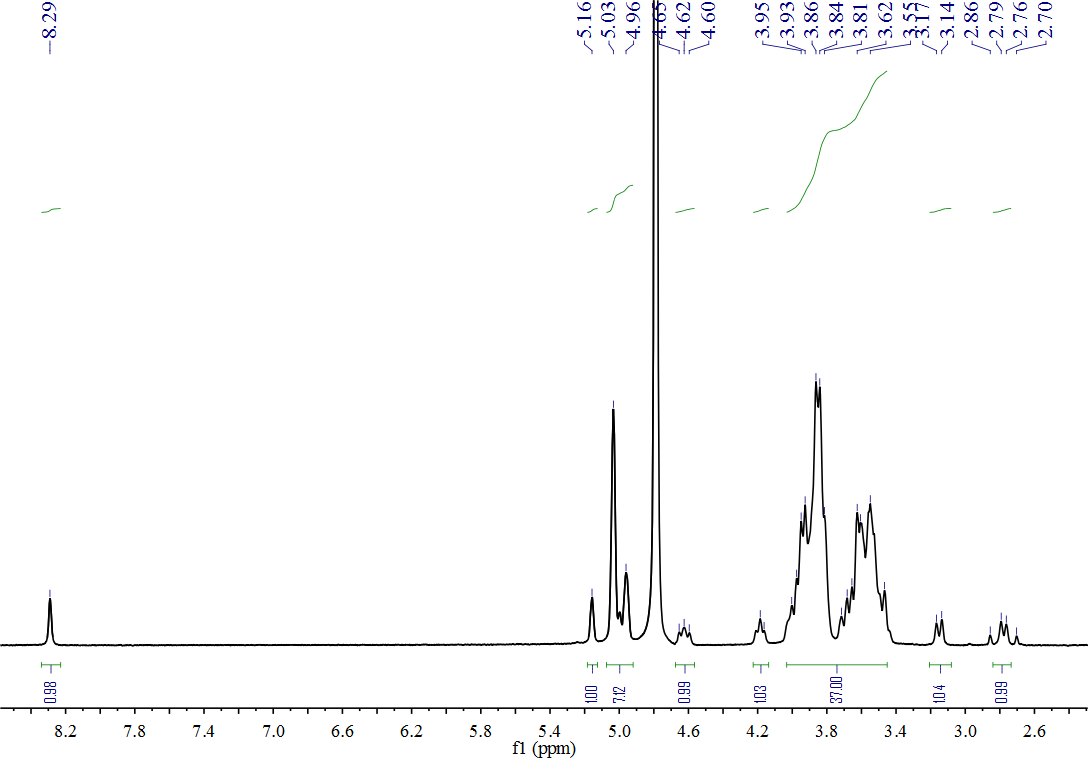


Figure S2. ^1^H NMR spectrum of **H_1_** in D_2_O, 20℃.


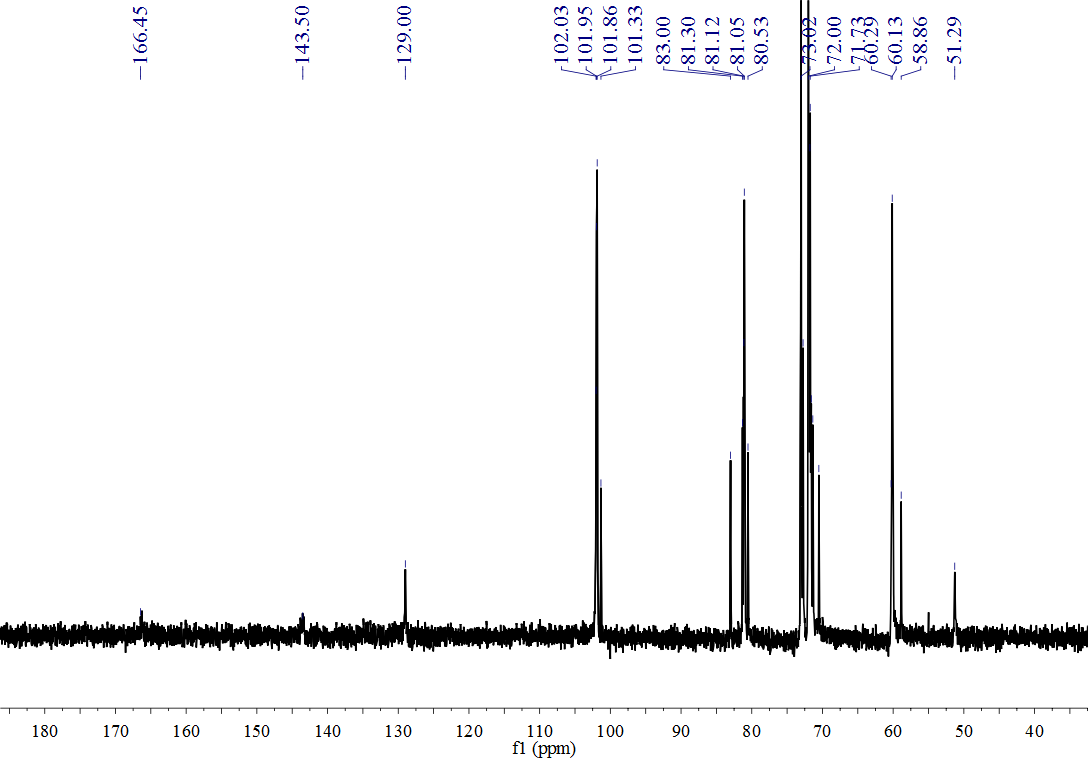


Figure S3. ^13^C NMR spectrum of **H_1_** in D_2_O, 20℃.

Figure S4. MALDI-TOF-MS spectrum of **H_1_.**


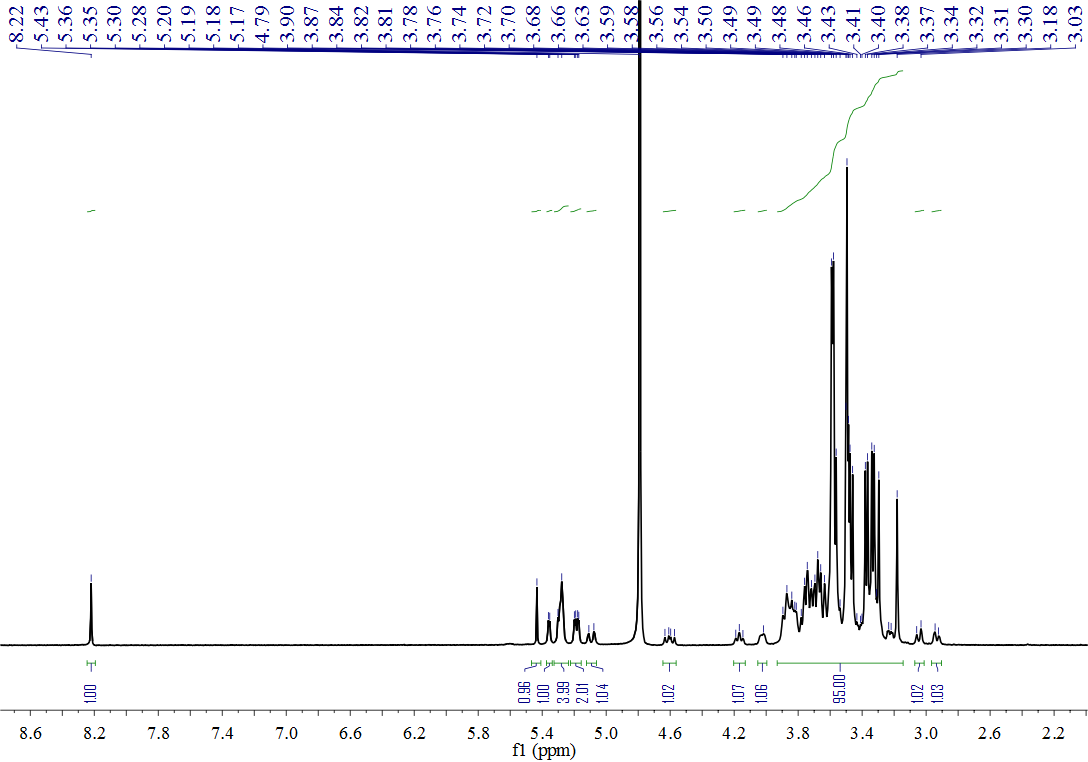


Figure S5. ^1^H NMR spectrum of **H_2_** in D2O, 20℃.


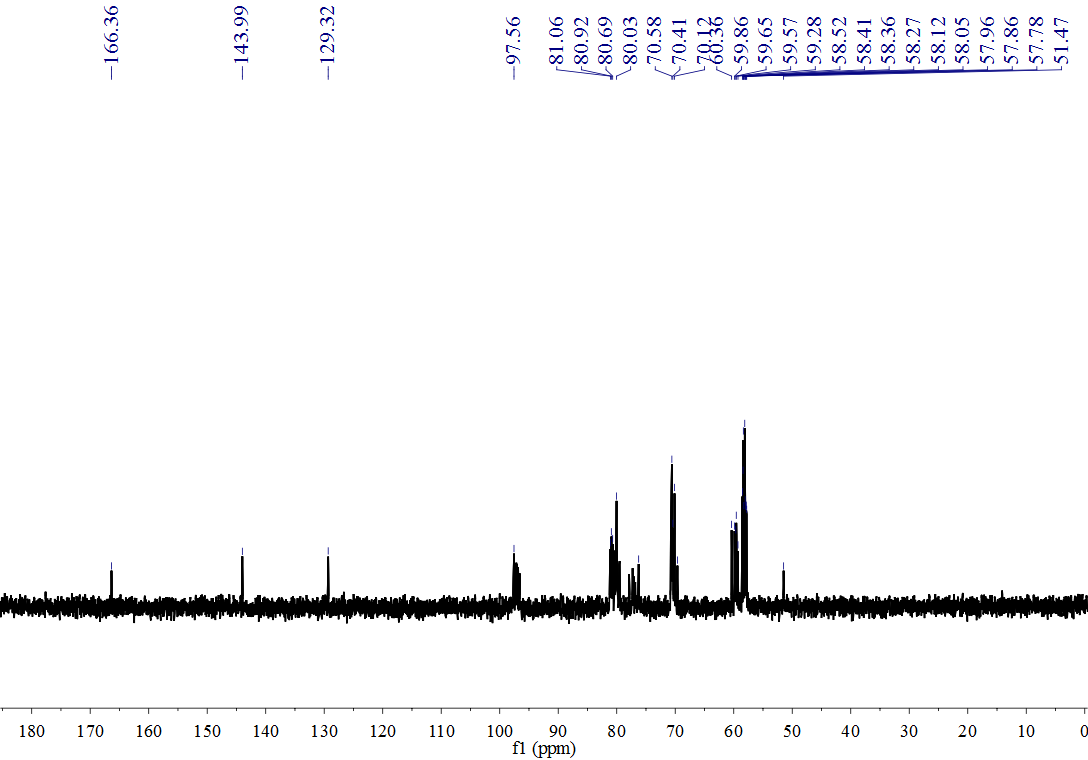


Figure S6. ^13^C NMR spectrum of **H_2_** in D2O, 20℃.
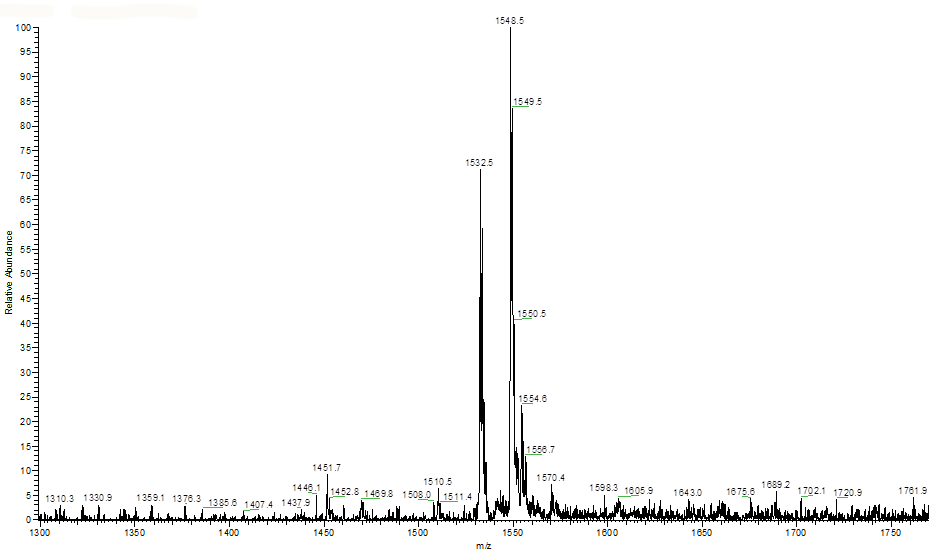


Figure S7. ESI-MS spectrum of **H_2_**.


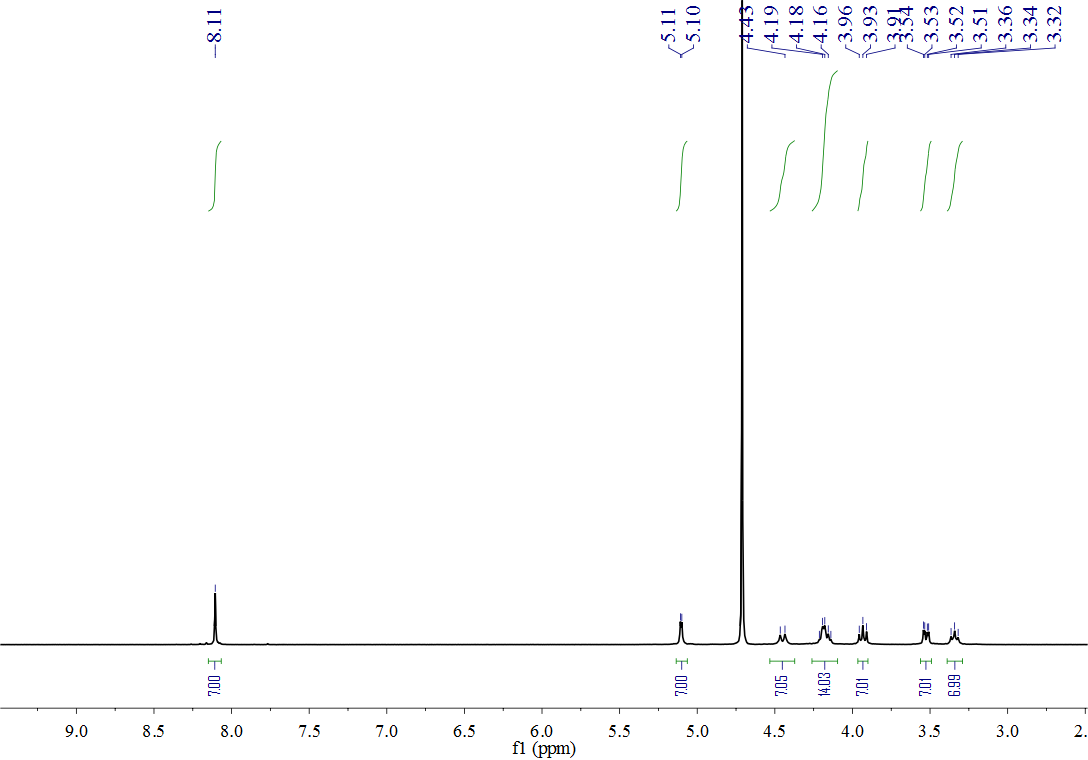


Figure S8. ^1^H NMR spectrum of **H_3_** in D2O, 20℃.


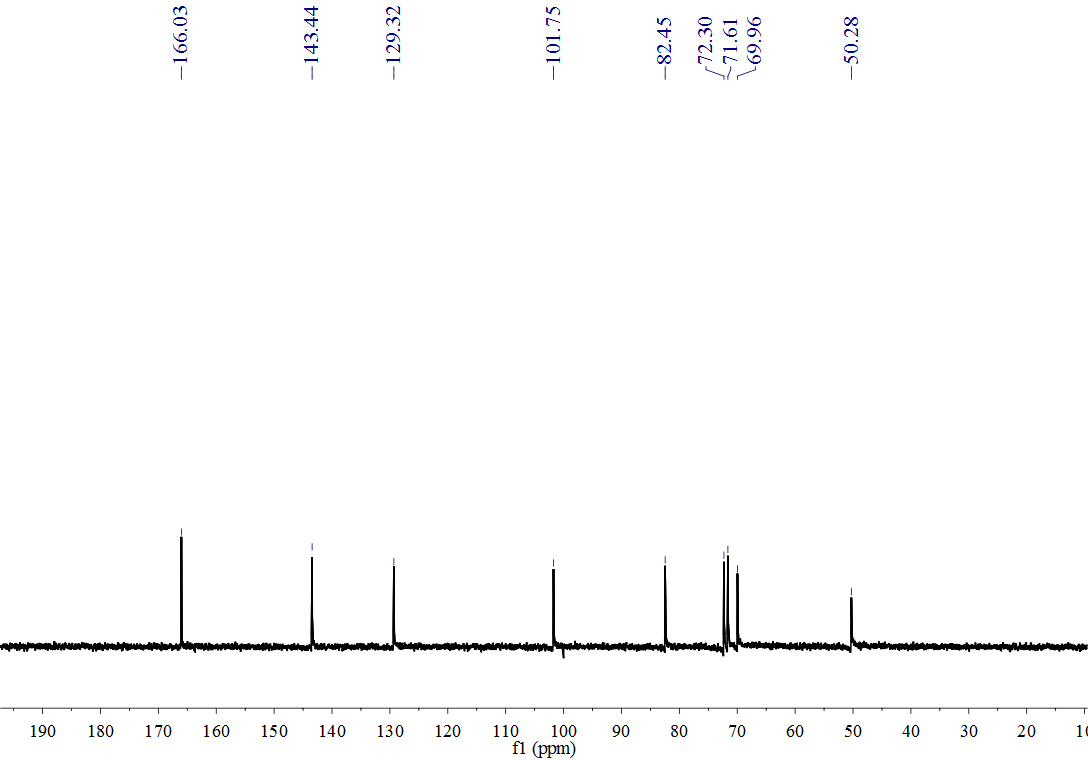


Figure S9. ^13^C NMR spectrum of **H_3_** in D2O, 20℃.


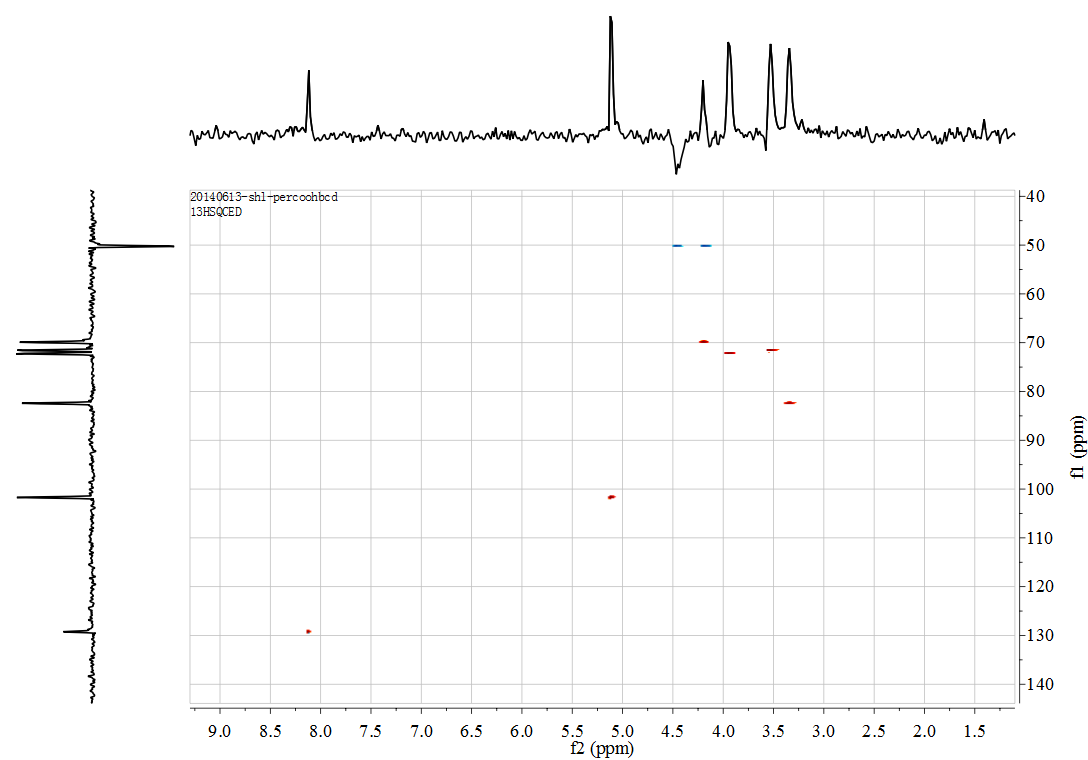


Figure S10. HSQC spectrum of **H_3_** in D2O, 20℃.


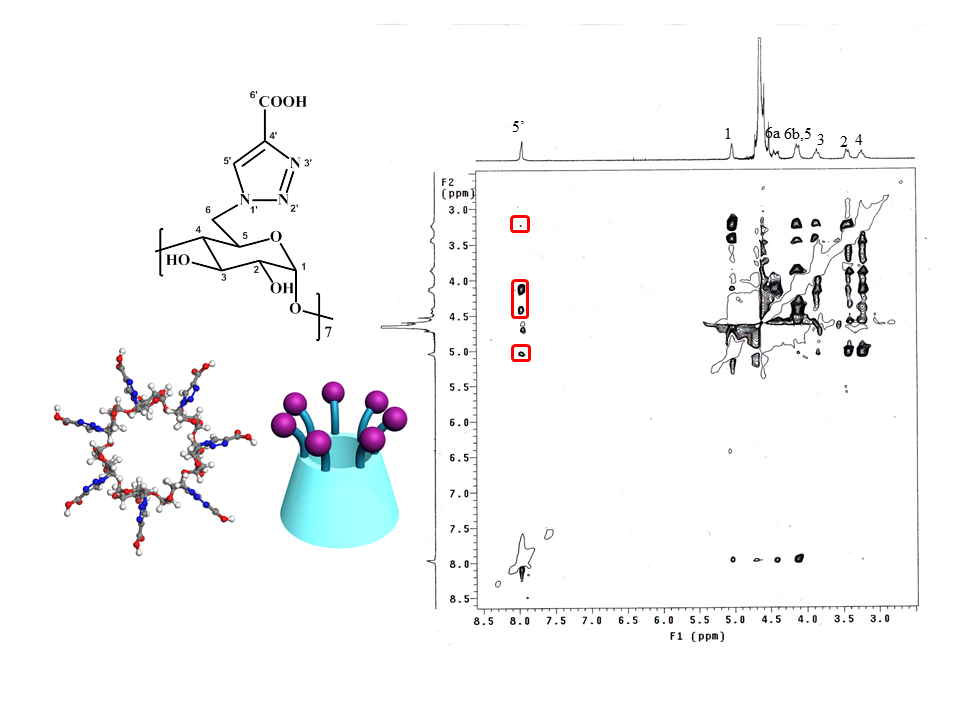


Figure S11. 2D ROESY spectrum of **H_3_** in D2O, 20℃.

Figure S12. MALDI-TOF-MS spectrum of **H3**


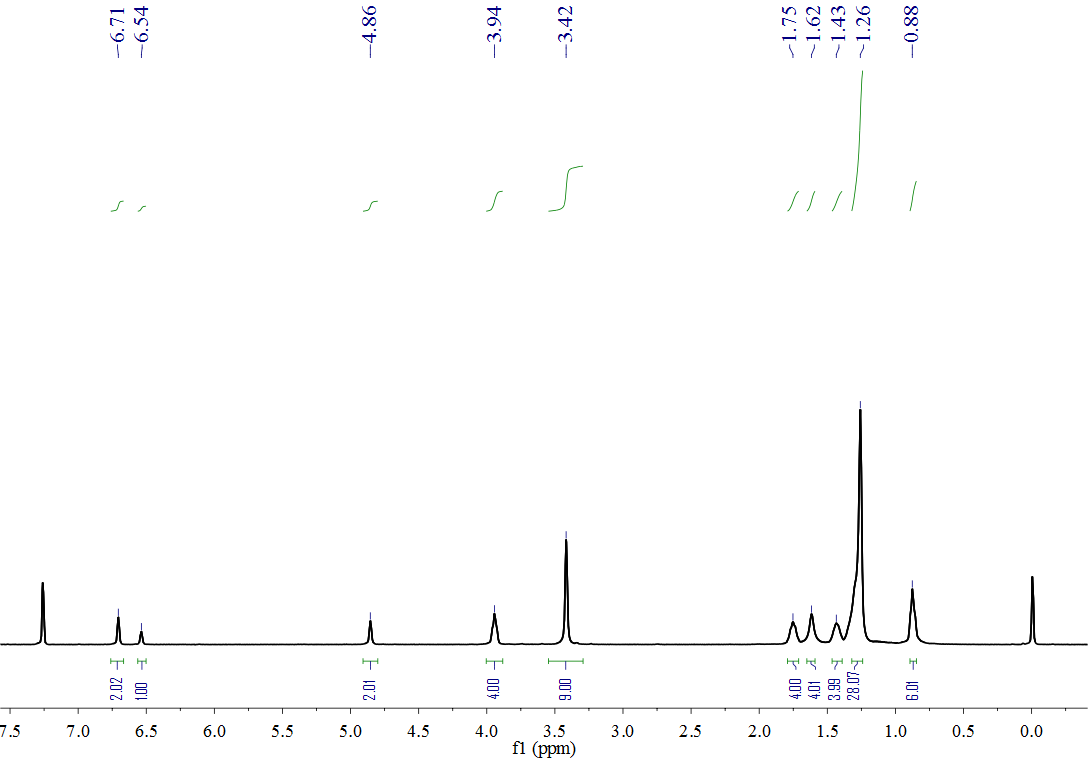


Figure S13. ^1^H NMR spectrum of **G** in CDCl_3_, 20℃.


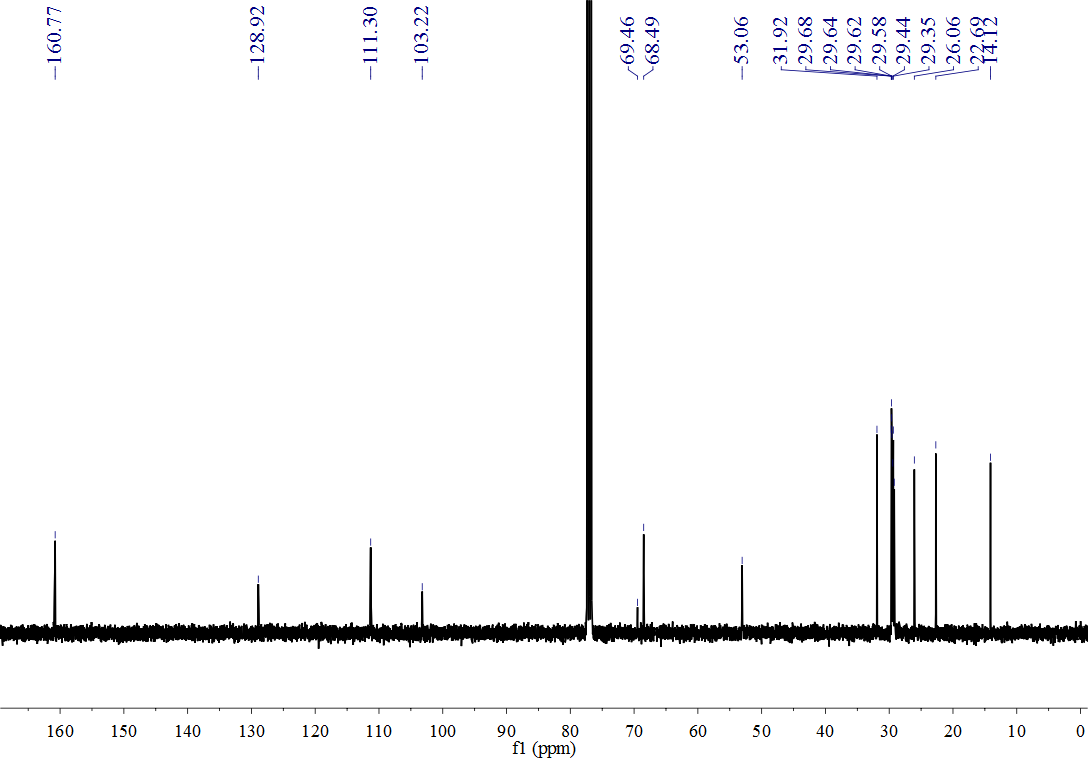


Figure S14. ^13^C NMR spectrum of **G** in CDCl_3_, 20℃.


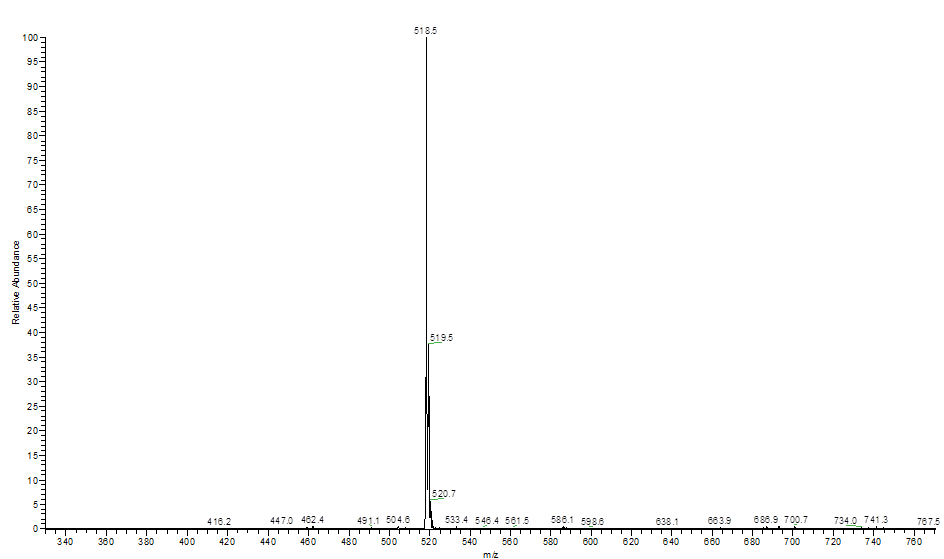


Figure S15. ESI-MS spectrum of **G**


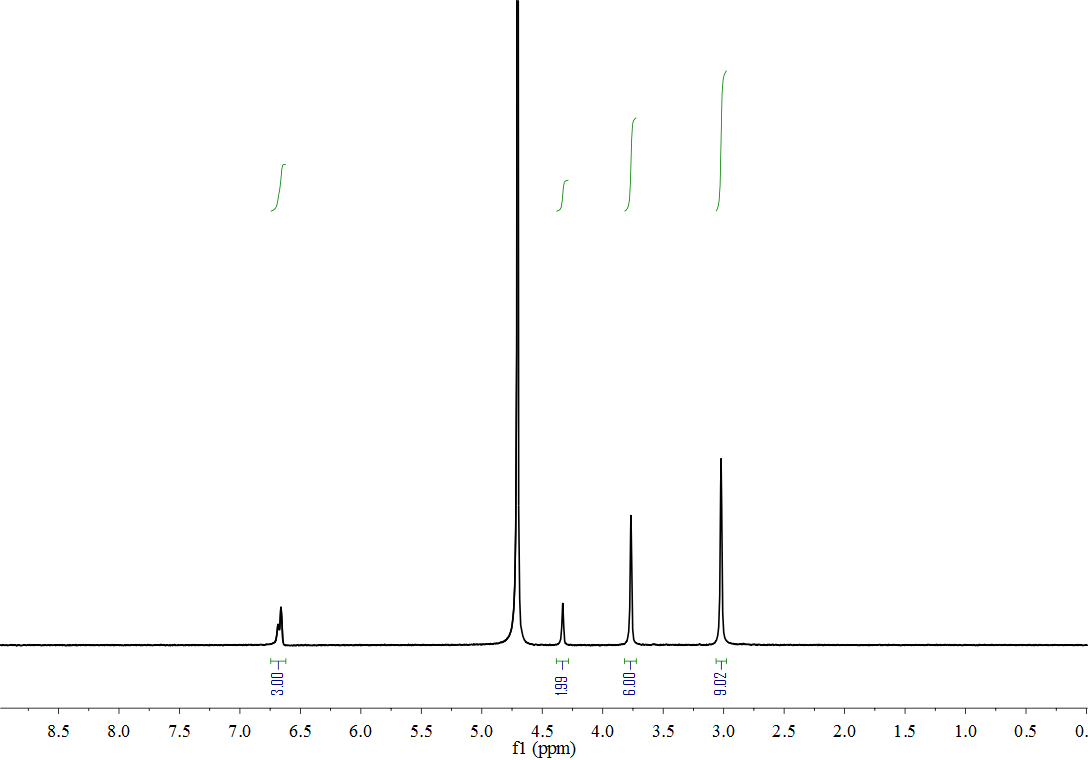


Figure S16. ^1^H NMR spectrum of **G_m_** in D2O, 20℃.


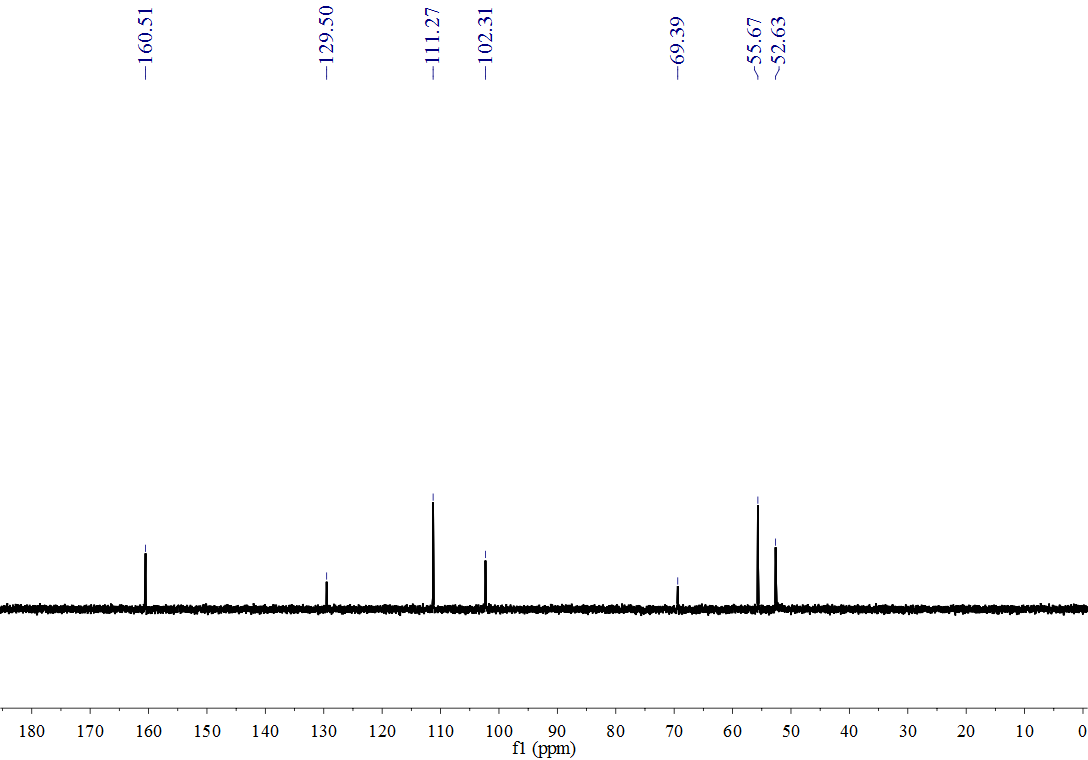


Figure S17. ^13^C NMR spectrum of **G_m_** in D2O, 20℃.


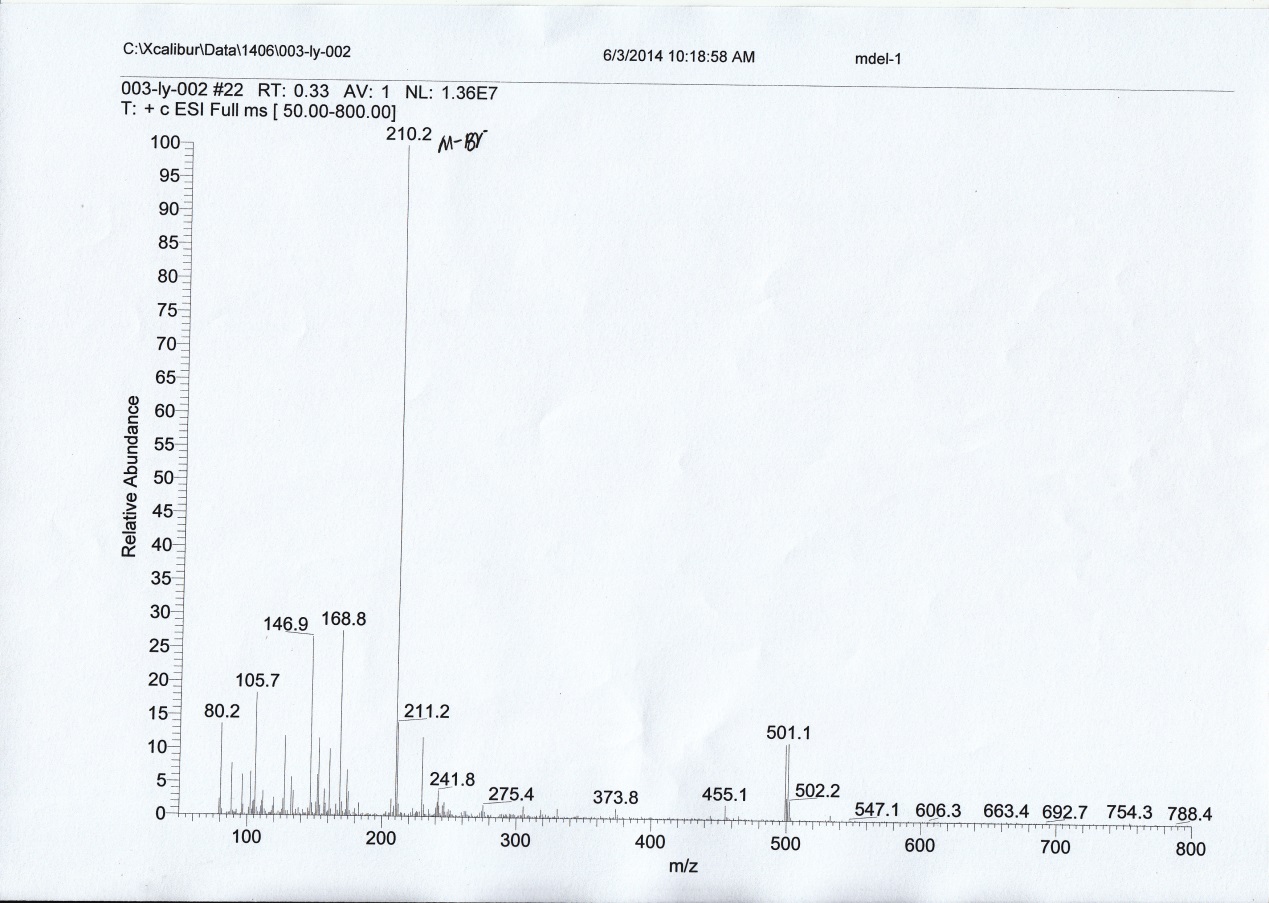


Figure S18. ESI-MS spectrum of **G_m_**


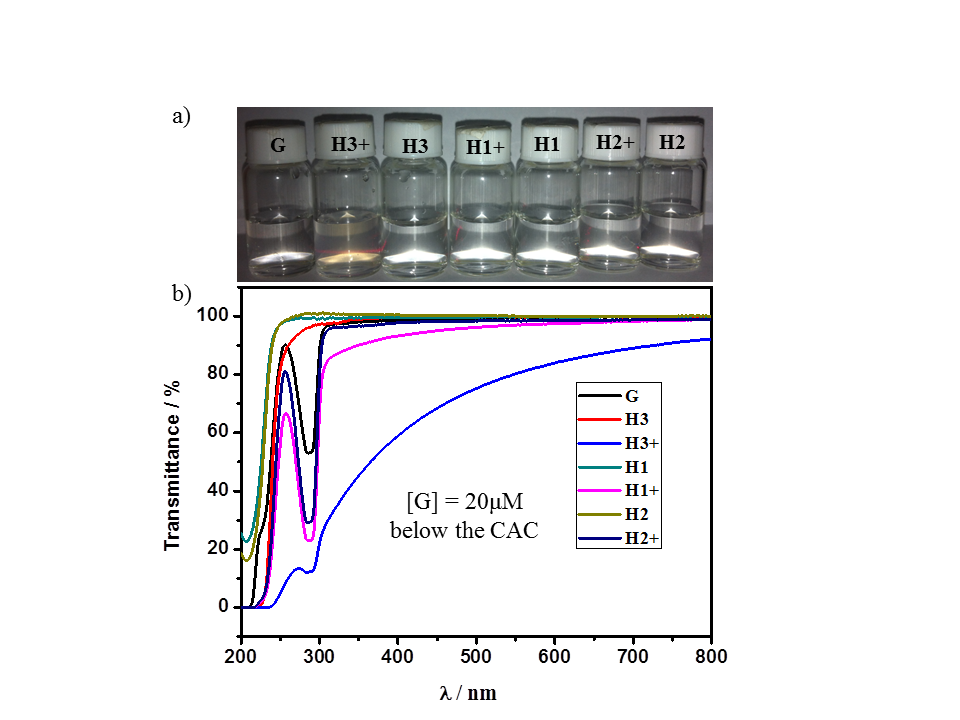


Figure S19. a) Tyndall effect, b) Transmittance spectra of G, H1, H2, H3 and their mixture with G respectively.


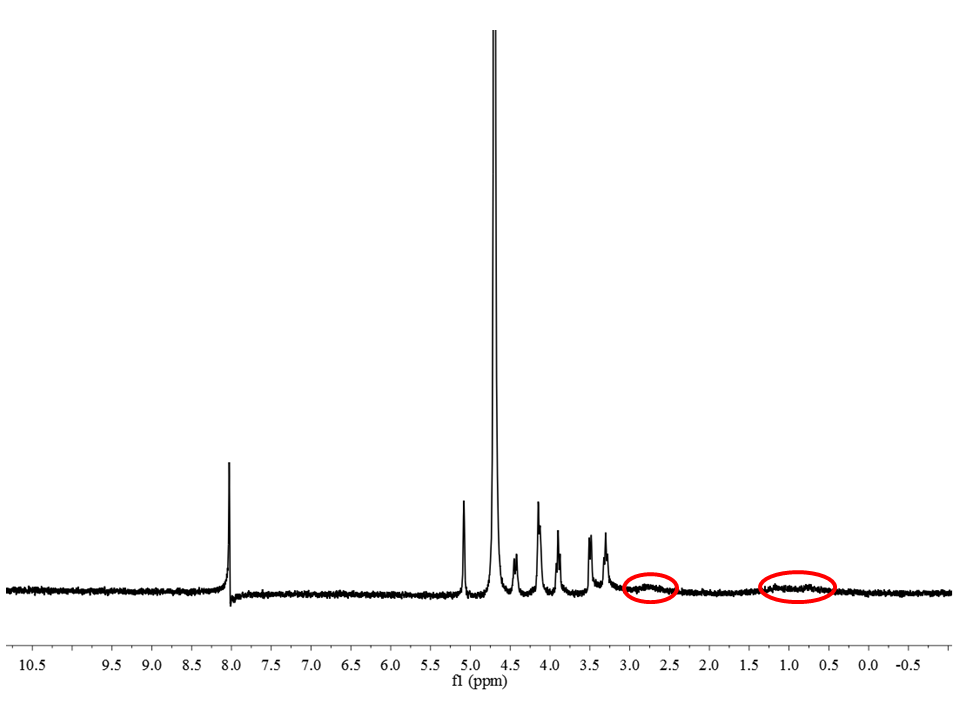


Figure S20. ^1^H NMR of **H_3_**@**G** in D2O, 20℃.


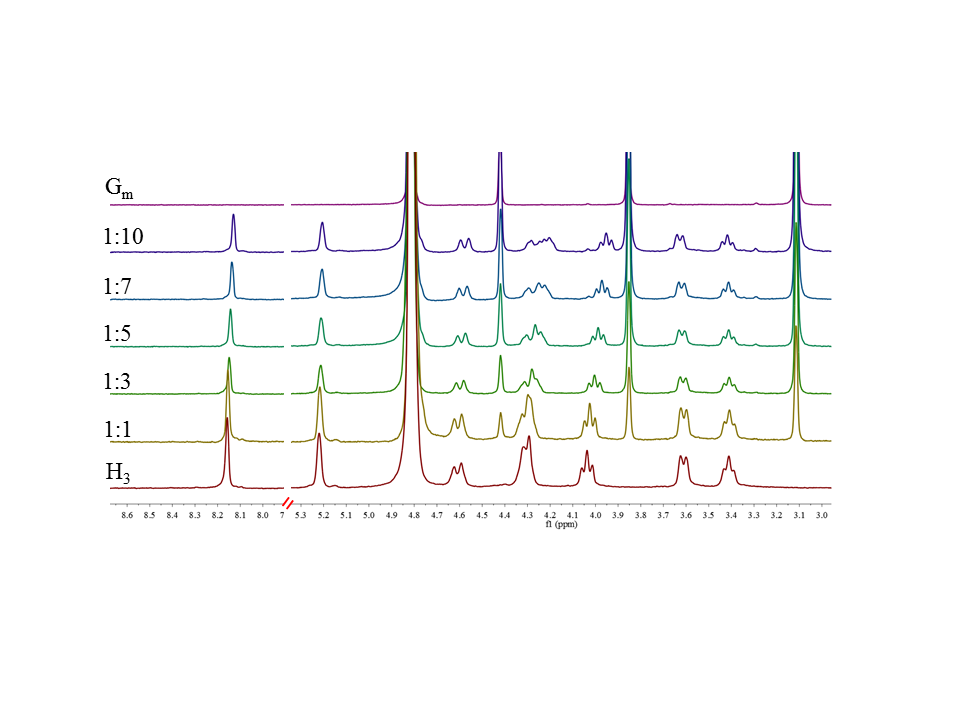


Figure S21. ^1^H NMR of **H_3_** with various ratio of **G_m_** in D2O, 20℃.


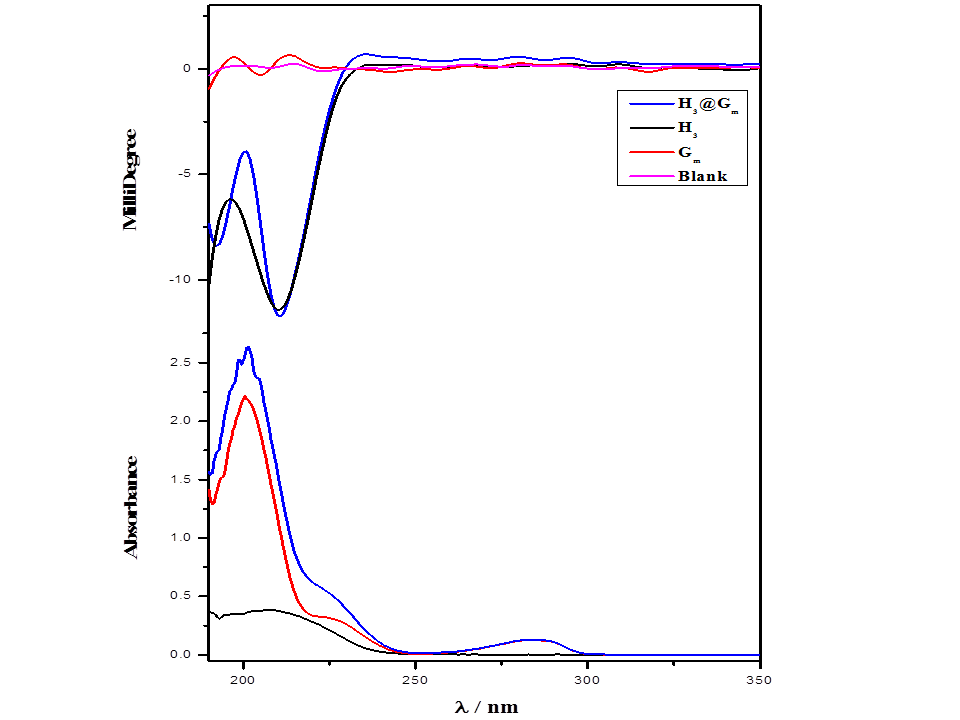


Figure S22. UV-Vis and CD spectra of **H_3_**, **G_m_** and **H_3_**@**G_m_** in aqueous solution pH=7.0, 25℃.


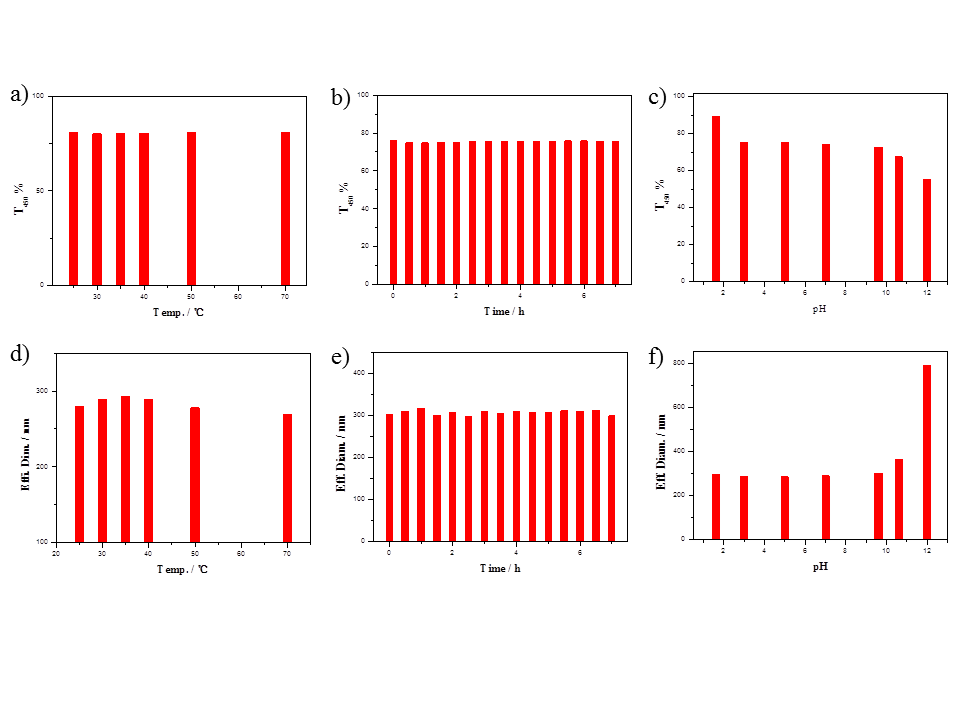


Figure S23. The stability of the assembly **H_3_**@**G** to temperature, time and pH determined by transmittance (a), b), c)) and DLS (d), e), f)).


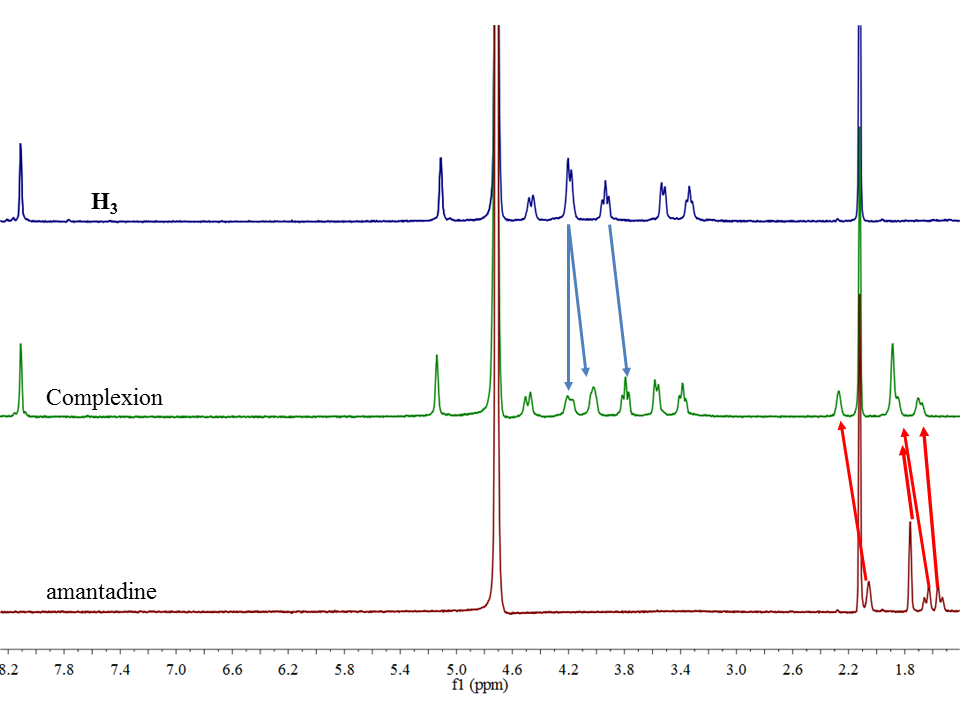


Figure S24. ^1^H NMR spectra of **H_3_**, amantadine and their inclusion complex in D2O, 20℃.


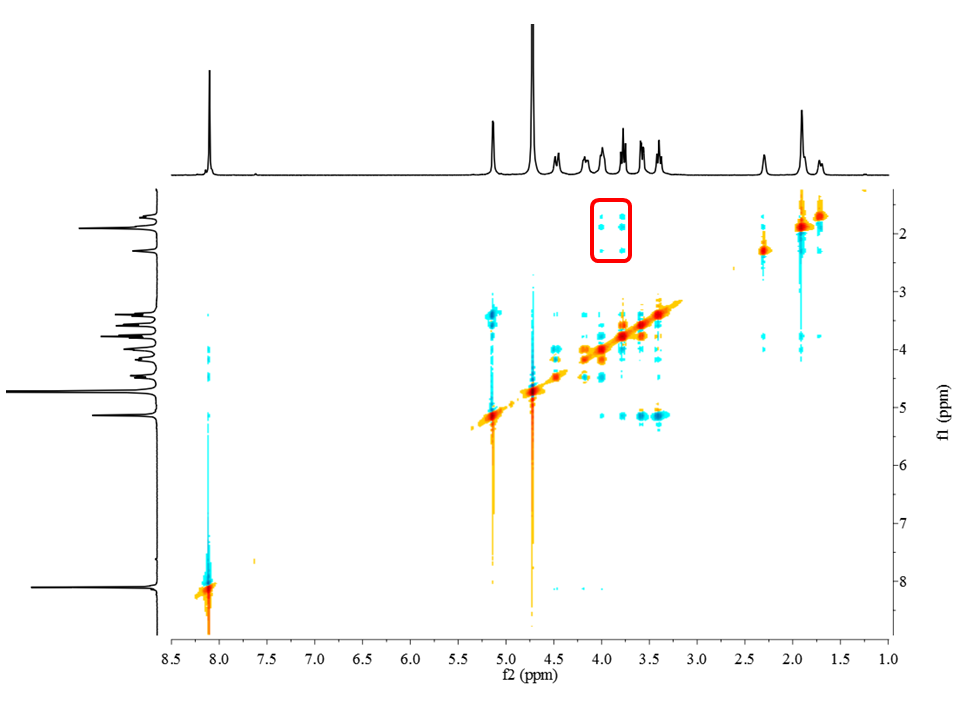


Figure S25. 2D ROESY spectrum of **H_3_**@Ama inclusion complex in D_2_O, 20℃.


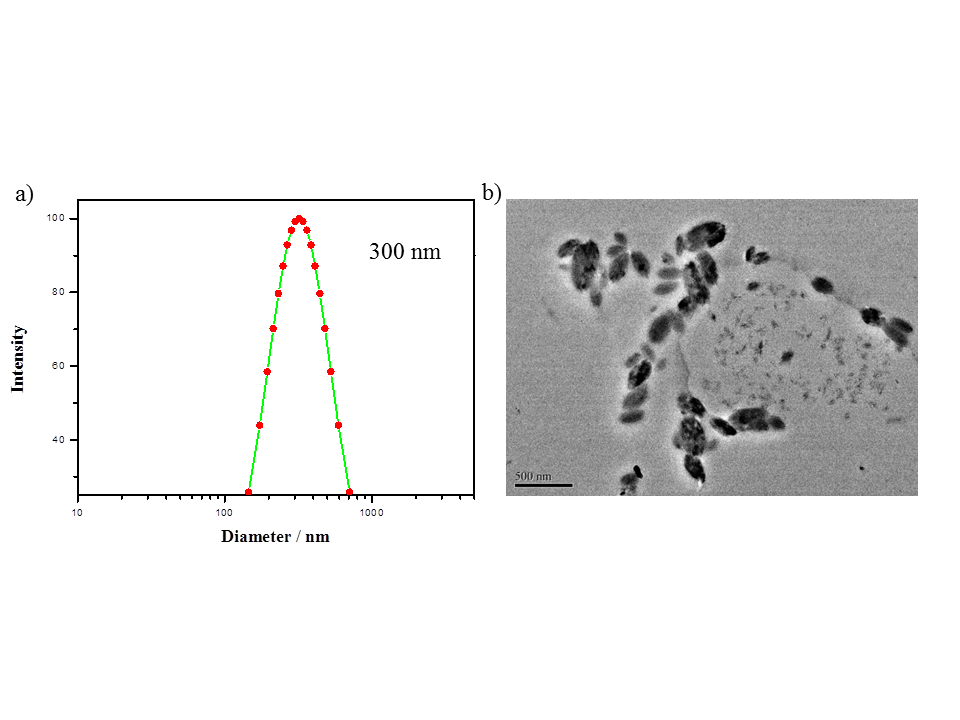


Figure S26. a) DLS and b) TEM image of the assembly **H_3_**@Ama@**G**.
